# Improved secretion of glycoproteins using an N-glycan-restricted passport sequence tag recognized by cargo receptor

**DOI:** 10.1038/s41467-020-15192-1

**Published:** 2020-03-13

**Authors:** Hirokazu Yagi, Maho Yagi-Utsumi, Rena Honda, Yusaku Ohta, Taiki Saito, Miho Nishio, Satoshi Ninagawa, Kousuke Suzuki, Takahiro Anzai, Yukiko Kamiya, Kazuhiro Aoki, Mahito Nakanishi, Tadashi Satoh, Koichi Kato

**Affiliations:** 1 Graduate School of Pharmaceutical Sciences, Nagoya City University, 3-1 Tanabe-dori, Mizuho-ku, Nagoya, 467-8603 Japan; 2 Exploratory Research Center on Life and Living Systems (ExCELLS), National Institutes of Natural Sciences, 5-1 Higashiyama, Myodaiji, Okazaki, 444-8787 Japan; 3 Institute for Molecular Science, National Institutes of Natural Sciences, 5-1 Higashiyama, Myodaiji, Okazaki, 444-8787 Japan; 4 School of Physical Science, SOKENDAI (The Graduate University for Advanced Studies), 5-1 Higashiyama, Myodaiji, Okazaki, 444-8787 Japan; 5 National Institute for Basic Biology, National Institutes of Natural Sciences, 5-1 Higashiyama, Myodaiji, Okazaki, 444-8787 Japan; 6 Biotechnology Research Institute for Drug Discovery, National Institute of Advanced Industrial Science and Technology (AIST), 1-1-1 Higashi, Central 5, Tsukuba, Ibaraki, 305-8565 Japan

**Keywords:** Biochemistry, Carbohydrates, Glycobiology, Proteins, Structural biology

## Abstract

MCFD2 and ERGIC-53, which are the products of causative genes of combined factor V and factor VIII deficiency, form a cargo receptor complex responsible for intracellular transport of these coagulation factors in the early secretory pathway. In this study, using an NMR technique, we successfully identified an MCFD2-binding segment from factor VIII composed of a 10 amino acid sequence that enhances its secretion. This prompted us to examine possible effects of attaching this sequence to recombinant glycoproteins on their secretion. We found that the secretion level of recombinant erythropoietin was significantly increased simply by tagging it with the passport sequence. Our findings not only provide molecular basis for the intracellular trafficking of coagulation factors and their genetic deficiency but also offer a potentially useful tool for increasing the production yields of recombinant glycoproteins of biopharmaceutical interest.

## Abstract (web summary)

The secretion of blood coagulation factor V and factor VIII (FV and FVIII) are driven by secretion factors ERGIC-53 and MCDF2. Here, the authors report a 10 amino acid motif from FVIII that can enhance secretion of another glycoprotein erythropoietin (EPO).

## Introduction

Recombinant glycoproteins and their derivatives, as typified by erythropoietin (EPO), are widely used as biologics^1^. Currently, most of these biologics are produced using mammalian expression systems following optimization of the culture medium and bioreactor conditions to improve production yields^2–4^. Attempts to improve expression have included the design of expression vectors with stronger promoters, codon optimization, and signal sequence optimization. Here we propose an alternative approach to increase the production yield of recombinant proteins by improving the efficiency of their intracellular trafficking in the early secretory pathway. The key component of our strategy is tagging of the target glycoprotein with a specific sequence derived from blood coagulation factors, which are recognized by a cargo receptor complex recycling the endoplasmic reticulum (ER) and the ER–Golgi intermediate compartment (ERGIC)^5,6^.

In the early secretory pathway, sophisticated mechanisms exist for the selective sorting and transport of specific secretory proteins as cargos, whereas certain abundant proteins are translocated via bulk flow without specific export signals^7^. The 53-kDa ERGIC marker protein, ERGIC-53 (or LMAN1), and its binding partner, multiple coagulation factor deficiency 2 (MCFD2), mediate the secretion of blood coagulation factor V (FV) and factor VIII (FVIII)^8–11^. These two proteins are known to be the causative gene products of combined deficiency of FV and FVIII (F5F8D), an autosomal recessive bleeding disorder in which the plasma levels of these coagulation factors are diminished^12–14^. Cumulative evidence has revealed that ERGIC-53 possesses a luminal carbohydrate recognition domain (ERGIC-53^CRD^) that binds the high-mannose-type glycans displayed on cargo glycoproteins in a pH-dependent manner^9,10,15,16^. The functional role of MCFD2, however, remains to be elucidated, although this protein harbors a calmodulin-like EF-hand domain and its putative orthologs have been identified in vertebrates and invertebrates prior to the evolutionary emergence of the blood coagulation system^11,17^. Our previous structural data indicated that the EF-hand motifs of MCFD2 undergo a conformational change upon interaction with ERGIC-53^CRD^ ^18^. This prompted us to hypothesize that MCFD2 is allosterically activated via interaction with ERGIC-53^CRD^ and thereby becomes able to bind a specific polypeptide segment contained in the cargo glycoproteins. Consequently, MCFD2 captures the glycoproteins in cooperation with ERGIC-53, which binds to their carbohydrate moieties. In the present study, we therefore searched for and identified an MCFD2-binding motif in FVIII and addressed the functional relevance of this binding motif in its secretion. Furthermore, we investigated the fusion of this motif to recombinant EPO to determine whether it would improve EPO expression levels by acting as a “passport” along the early secretory pathway, as mediated by the ERGIC-53/MCFD2 complex.

## Results and discussion

**Fig. 1:**
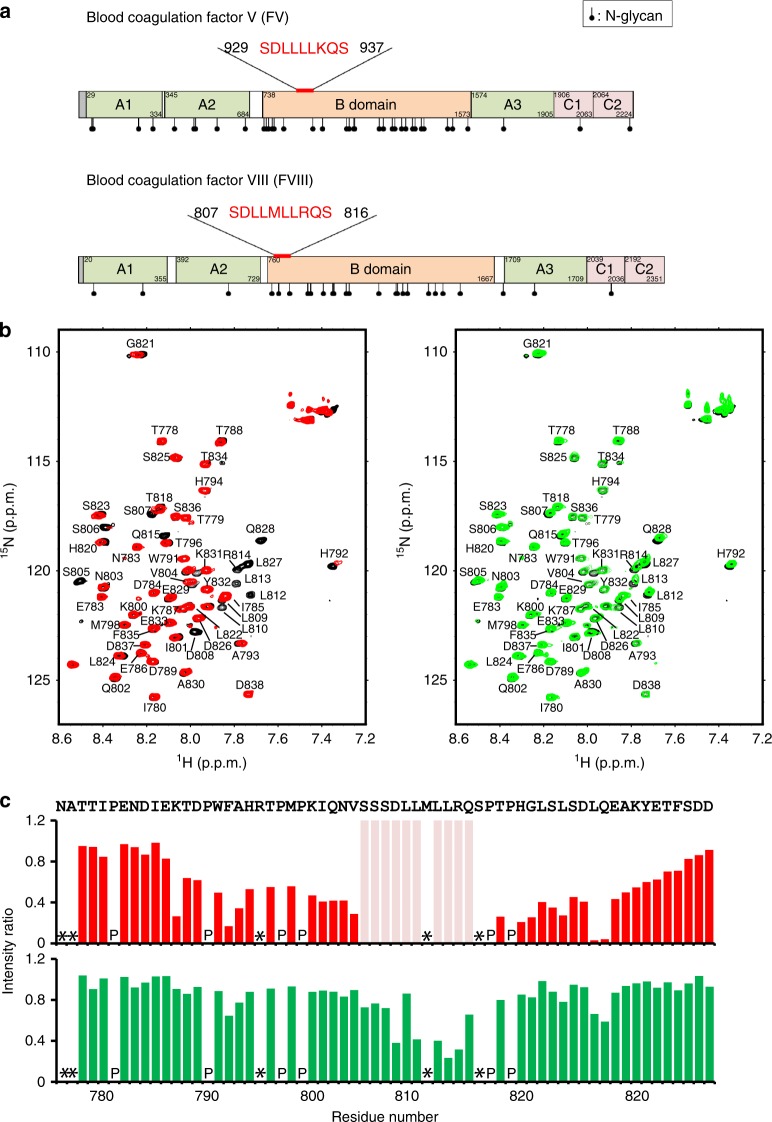
The N-terminal segment of the FVIII B domain interacts with MCFD2/ERGIC-53^CRD^. **a** Schematic representation of the domains in factors V and VIII. Conserved domains and the range of amino acid residues comprising each domain are indicated. The SDLL(-/M)LL(K/R)QS consensus motifs identified in FV and FVIII are highlighted in red. *N*-linked glycosylation sites as annotated in the UniProt database (P12259 and P00451), are represented. **b** ^1^H–^15^N heteronuclear single quantum coherence (HSQC) spectra of the ^15^N-labeled FVIII-derived peptide (Asn776–Asp838) alone (black) or in the presence of one molar equivalent of MCFD2/ERGIC-53^CRD^ (left) or MCFD2 (right). The ^15^N-labeled FVIII-derived peptide (0.1 mM) was dissolved in 20 mM MES (pH 6.0) containing 10 mM CaCl_2_, 150 mM NaCl, and 10% (v/v) D_2_O. NMR spectra were acquired at 283 K. **c** Plots of the intensity ratios of the backbone amide peaks of the FVIII-derived peptide with versus without MCFD2/ERGIC-53^CRD^ (upper) or MCFD2 only (lower). Pink bars indicate residues whose NMR peaks were undetectable due to extreme broadening upon addition of MCFD2/ERGIC-53^CRD^. In the profiles of intensity ratio, residues for which the ^1^H–^15^N HSQC peaks could not be observed because of peak overlapping and/or broadening were denoted with asterisks, whereas proline residues were denoted with “P”.

**Fig. 2:**
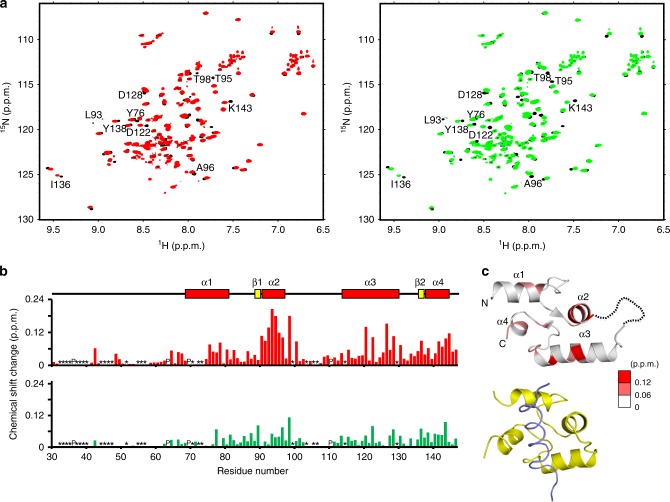
MCFD2 interacts with FVIII through the canonical ligand-binding site of EF-hand proteins. **a** ^1^H–^15^N HSQC spectra of [^15^N]MCFD2 with five molar equivalents of the ERGIC-53^CRD^-derived peptide (RRFEYKYSFKGPH) in the presence (red) and absence (black) of two molar equivalents of the FVIII-derived peptide (DPPSDLLLLKQSNSSKILVGRWHLASEK) (left). ^1^H–^15^N HSQC spectra of [^15^N]MCFD2 in the presence (green) and absence (black) of two molar equivalents of the FVIII-derived peptide (right). Proteins were dissolved in 20 mM MES (pH 6.0) containing 10 mM CaCl_2_, 150 mM NaCl, and 10% (v/v) D_2_O. NMR spectra were acquired at 303 K. **b** NMR chemical shift changes observed for MCFD2 in the presence (upper) and absence (lower) of the ERGIC-53^CRD^-derived peptide upon the addition of the FVIII-derived peptide. In the NMR perturbation profiles, residues for which the ^1^H–^15^N HSQC peaks could not be observed because of peak overlapping and/or broadening were denoted with asterisks, whereas proline residues were denoted with “P.” The observed chemical shift perturbations were quantified as Δ*δ* = [(Δ*δ*_H_)^2^ + (Δ*δ*_N_/5)^2^]^1/2^, where Δ*δ*_H_ and Δ*δ*_N_ were the observed chemical shift changes for ^1^H and ^15^N, respectively. **c** The perturbed residues were mapped on a ribbon model of MCFD2 (upper). The dotted line indicates a disordered loop. A superimposed ligand-bound calmodulin complex (PDB code: 1CFF) is also shown (lower). The calmodulin and Ca^2+^-pump peptide ligand structures are colored in yellow and slate, respectively.

**Fig. 3:**
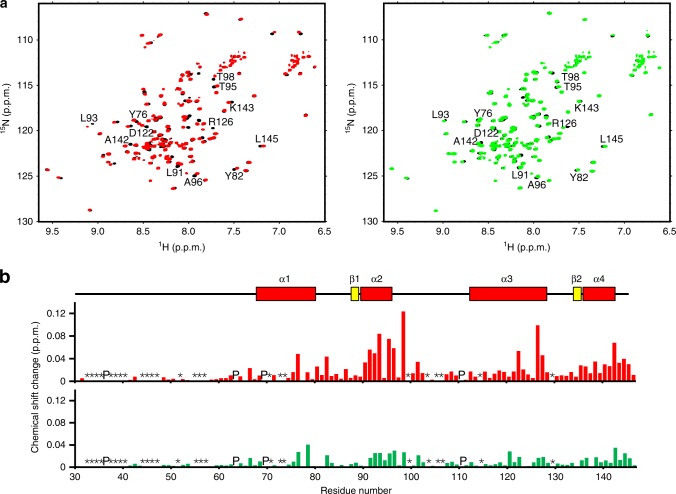
NMR characterization of the interaction of MCFD2 with the FV-derived peptide. **a** ^1^H–^15^N HSQC spectra of [^15^N]MCFD2 with five molar equivalents of the ERGIC-53^CRD^-derived peptide (RRFEYKYSFKGPH) in the presence (red) and absence (black) of two molar equivalents of the FV peptide (DPPSDLLLLKQSNSSKILVGRWHLASEK) (left). ^1^H–^15^N HSQC spectra of [^15^N]MCFD2 in the presence (green) and absence (black) of two molar equivalents of the FV peptide (right). The synthetic FV-derived peptide was purchased from AnyGen Co., Ltd., and dissolved in 100% dimethyl sulfoxide-*d*6 (DMSO-*d*6). Proteins were dissolved in 20 mM MES (pH 6.0) containing 10 mM CaCl_2_, 150 mM NaCl, 2% (v/v) DMSO-*d*6, and 10% (v/v) D_2_O. NMR spectra were acquired at 303 K. **b** NMR chemical shift changes observed for MCFD2 in the presence (upper) and absence (lower) of the ERGIC-53^CRD^-derived peptide upon addition of the FV-derived peptide. In the NMR perturbation profiles, definitions of asterisks, “P,” and chemical shift change are the same as those in Fig. 2.

Previous reports suggest that FVIII interacts with the cargo receptor complex through its B domain^19^. We focused on the N-terminal segment (residues 760–985) of the B domain because deletion of this segment influences the secretion level of FVIII^20^. We found that human FV and FVIII share similar sequences in their B domains, that is, SDLLMLLRQS at residues 807–816 in FVIII versus SDLLLLKQS at residues 929–937 in FV (Fig. 1a). This suggests that SDLL(-/M)LL(K/R)QS is a common motif recognized by MCFD2 in complex with ERGIC-53. Nuclear magnetic resonance (NMR) data obtained using a ^15^N-labeled FVIII fragment corresponding to residues 776–838 indicated significant attenuation in peak intensities for the region spanning residues 806–819 upon addition of the MCFD2/ERGIC-53^CRD^ complex, demonstrating that the putative motif is indeed involved in interaction with the cargo receptor complex (Fig. 1b, c). From the titration data, the dissociation constant for the interaction between the FVIII-derived peptide and MCFD2/ERGIC-53^CRD^ was calculated to be 1.1 ± 0.1 × 10^−5^ M. Notably, this interaction was much less pronounced in the absence of ERGIC-53^CRD^ (1.2 ± 0.4 × 10^−4^ M). We also performed NMR experiments using ^15^N-labeled MCFD2. We have already reported that the tridecapeptide corresponding to the MCFD2-binding segment Arg44–His56 in ERGIC-53^CRD^ induces widespread chemical shift changes of MCFD2 because of its conformational transition^18^. The FVIII-derived peptide Asn776–Asp838 in the present study also induced chemical shift changes of MCFD2, which presented as markedly enhanced spectral changes in the presence of the ERGIC-53-derived tridecapeptide (Fig. 2a, b). The perturbed residues were located in the α2 and α3 helices of MCFD2, which correspond to the canonical ligand-binding site of EF-hand proteins as observed in calmodulin^21^ (Fig. 2c). Our NMR experiments confirmed that the peptide fragment comprising residues 926–953 of FV, which also contains the putative MCFD2-binding motif, interacts with the EF-hand helices of MCFD2 in an ERGIC-53^CRD^-dependent manner (Fig. 3). These data indicate that MCFD2 interacts with the SDLL(-/M)LL(K/R)QS motif in the B domains of FV and FVIII through its EF-hand helices, which are allosterically activated by ERGIC-53^CRD^. Thus, the identified MCFD2-binding motif is highly conserved in FV and FVIII across mammalian species (Supplementary Fig. 1). A similar sequence TDLLQLLLPR is found in human galectin-3-binding protein, which is also secreted depending on MCFD2^22^.

**Fig. 4:**
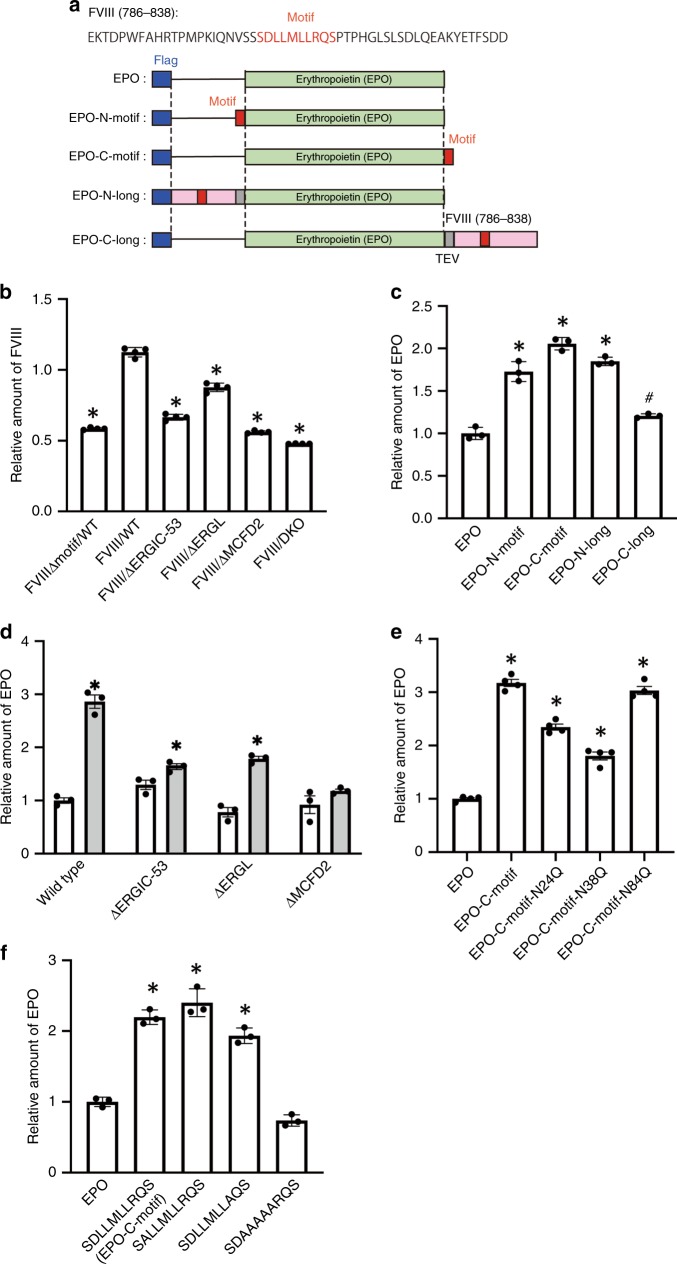
The effect of the MCFD2-binding motif on the secretion levels of erythropoietin. **a** Schematic representation of recombinant EPO proteins used in this study. The MCFD2-binding motif is colored red. The FVIII-derived peptide (Glu786–Asp838) [FVIII(786-838)], EPO, tandem flag tag peptide (Flag), and TEV protease site (TEV) sequences are represented in pink, light green, blue, and gray, respectively. In EPO-N-motif and EPO-C-motif, the MCFD2-binding motif was directly connected to the N or C terminus of EPO, respectively. In EPO-N-long and EPO-C-long, the FVIII-derived peptide was connected to the N or C terminus of EPO, respectively, with a TEV sequence insertion. **b** Deletion of the SDLLMLLRQS motif caused reduced secretion of FVIII from HCT116 cells. Wild-type FVIII and its motif-deleted mutant (FVIIIΔmotif) were expressed in HCT116 cells, and the secretion levels of FVIII antigen in the culture medium of wild-type cells (WT) as well as MCFD2 (ΔMCFD2), ERGIC-53 (ΔERGIC-53) and ERGIC-53/ERGL (ΔDKO) KO cells were determined with a commercial sandwich enzyme-linked immunosorbent assay. Error bars represent the standard error of the mean (SEM) (*n* = 4 independent transfections). Significant differences (*) were calculated compared with WT FVIII expression levels using two-tailed unpaired Student’s *t* test (*p* < 0.001). Transfection was normalized against controls transfected with the GFP expression vector. **c** Tagging with the SDLLMLLRQS motif caused increased secretion of EPO from HCT116 cells. **d** Secretion enhancement of EPO by its tagging with the motif was compromised in ΔMCFD2 or ΔERGIC-53 cells. The secretion levels of EPO and EPO-C-motif were represented by white and gray bars, respectively. **e** The effect of N-glycosylation on EPO-C-motif secretion from HCT116 cells was estimated using three kinds of N-glycan-deficient mutants (N24Q, N38Q, and N84Q). **f** The effect of alanine substitutions in the SDLLMLLRQS motif was determined in HCT116 cells. **c**–**f** The secretion level of EPO with or without the C-terminal SDLLMLLRQS motif was measured in the culture medium of WT as well as ΔMCFD2 and ΔERGIC-53 cells using an enzyme-linked immunosorbent assay. Error bars represent the SEM (*n* = 3 independent transfections). Significant differences (* and ^#^) were calculated compared with control EPO expression levels using two-tailed unpaired Student’s *t* test (*p* < 0.001 and *p* < 0.01, respectively). Transfection was normalized against controls transfected with the GFP expression vector.

To address the functional relevance of the interaction of the identified motif with MCFD2, we conducted secretion assays using FVIII-transfected HCT116 cells. Deletion of the MCFD2-binding motif from FVIII resulted in marked reduction in FVIII secretion, to a similar extent as that obtained by knocking out either MCFD2 or ERGIC-53 using CRISPR/Cas9-mediated mutagenesis (Fig. 4a, b). These results demonstrate the significant positive contribution of the MCFD2-binding motif to FVIII secretion. The secretion level of FVIII in the ERGIC-53-knockout (KO) cells was significantly higher in comparison with that in the MCFD2-KO cells, suggesting a compensation mechanism, most plausibly mediated by ERGIC-53-like protein, ERGL^23^, which share the MCFD2-binding sequence with ERGIC-53 (Supplementary Fig. 2). Indeed, our NMR data demonstrated that an ERGL-derived tridecapeptide corresponding to the MCFD2-binding sequence activated MCFD2 in terms of interaction with the FVIII-derived peptide (Supplementary Figs. 1 and 2). Consistent with this, FVIII secretion level was reduced in ERGL-KO cells to the same extent as that in the ERGIC-53-KO cells and synergistically reduced in ERGIC-53/ERGL double-KO cells (Fig. 4b). These data indicate that ERGL as well as ERGIC-53 allosterically activates MCFD2 and thereby cooperatively interacts with FVIII. Although the elementary interactions between ERGIC-53/ERGL and an FVIII glycan and between MCFD2 and its binding motif identified in this study are weak, their dual binding is likely to enhance the affinity of the cargo to be at physiologically relevant levels.

We next fused the MCFD2-binding motif to EPO to examine its possible effect on EPO secretion from EPO-transfected HCT116 cells (Fig. 4a). As expected, the secretion level of EPO was increased by tagging it with this motif, and the extent of this increase varied depending on the positioning of the motif sequence (Fig. 4c). The most effective enhancement was achieved by C-terminal fusion without additional spacer sequence. We further examined possible effects of the C-terminal tagging of the motif sequence to other recombinant glycoproteins on their secretion levels from HCT116 cells, that is, human α1-antitrypsin (AAT) and the soluble form of human Fcγ receptor III (sFcγRIII), which possess three and five N-glycosylation sites, respectively. The C-terminal tagging also upregulated the secretion of sFcγRIII but not AAT (Supplementary Fig. 4). The enhanced secretion of the recombinant EPO glycoprotein with the C-terminal motif (EPO-C-motif) was also observed in Expi293 and HEK293T cells (Supplementary Fig. 5). This improvement was largely compromised by knocking out either MCFD2, ERGIC-53, or ERGL (Fig. 4d) and negatively affected by deglycosylation of EPO (Fig. 4e). This indicated that the cargo receptor complex dually recognizes the N-glycans as well as the motif sequence tag displayed on EPO. The impact of deglycosylation depends on the N-glycosylation sites, suggesting that the spatial arrangement between the N-glycans and the MCFD2-binding motif is a determining factor in the recognition of cargos by the cargo receptor complex.

Both the secretion assay of EPO with alanine substitutions in the C-terminal tag and NMR interaction analysis indicated that the central leucine cluster rather than the flanking charged residues was crucial for MCFD2 binding and the consequent secretion of EPO (Fig. 4f and Supplementary Fig. 6). These data are consistent with those of a previous study^20^, which showed that the central leucine cluster, rather than the following sequence, significantly contributes to FVIII secretion. We confirmed that the replacement of the leucine cluster with successive alanine residues resulted in significant reduction in the levels of FVIII secretion from HCT116 and FV secretion from COS-7 cells (Supplementary Fig. 7).

**Fig. 5:**
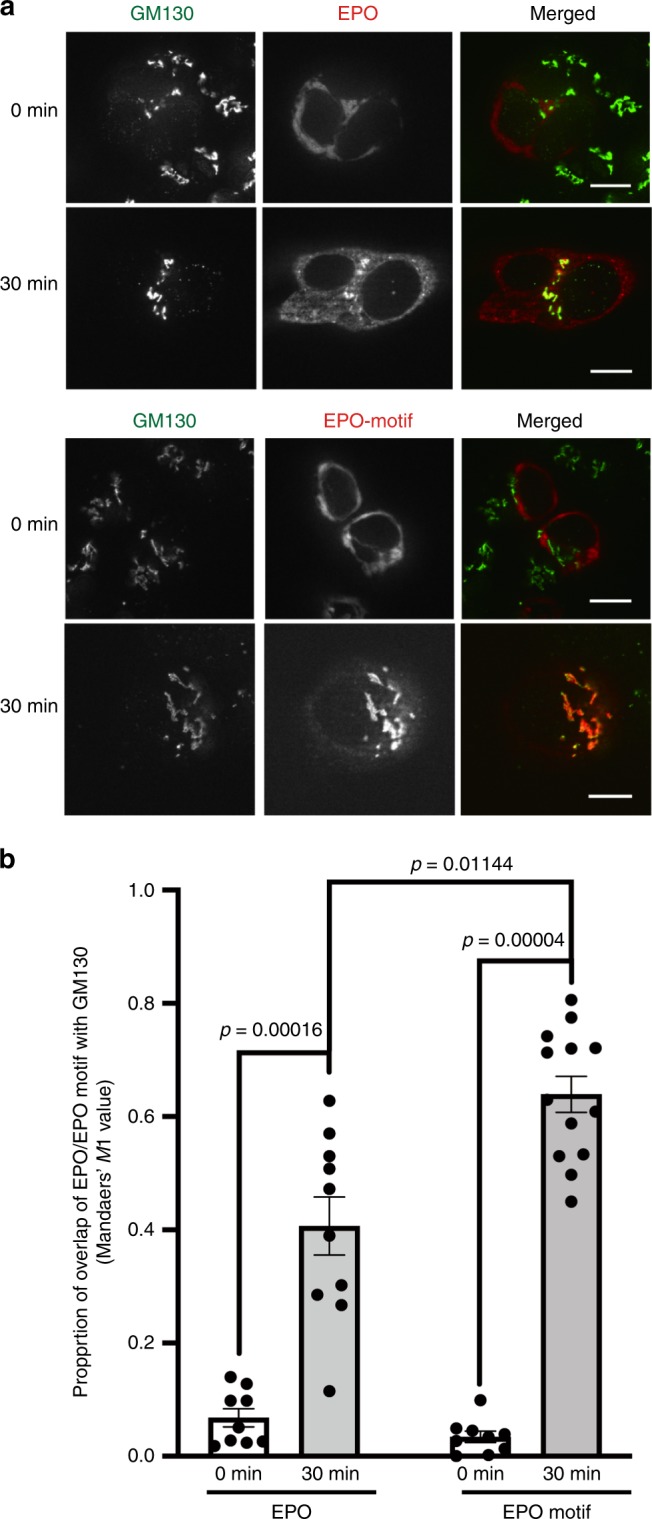
Effect of the passport sequence tagging on the ER-to-Golgi transport of EPO. **a** RUSH imaging of EPO and EPO-motif expressed in HCT116. Before the addition of biotin (time 0), both EPO and EPO-motif exhibited a typical ER localization. At 30 min after biotin addition, EPO was localized in the Golgi to varying extents depending on the C-terminal motif. GM130 was used as a *cis*-Golgi marker. Bar, 10 µm. Consistent data were obtained from two independent experiments. **b** Quantification of the trafficking efficiency to the Golgi complex in HCT116 cells. Colocalized fractions of EPO and EPO-motif with GM130 were quantified based on Manders’ *M*1 coefficient^32^. Data are means of at least nine cells. Error bars show SEM. Statistical significance (*p*) was assessed by Wilcoxon’s and Mann–Whitney tests with two tailed.

These raise the possibility that interactions of the cargo glycoproteins with the MCFD2/ERGIC-53 complex facilitate vesicular transport from the ER to the ERGIC, consequently increasing their expression level. We examined the effects of the passport sequence tagging of EPO on the anterograde traffic from the ER to the Golgi using the RUSH (retention using selective hooks) system^24^. The RUSH data indicated that the trafficking efficiency of EPO was enhanced by tagging it with the passport sequence (Fig. 5).

The present study demonstrated the functional importance of the MCFD2-binding motif as a passport sequence valid for anterograde transport of FV and FVIII in the early secretory pathway. This provides a molecular basis for the pathology of F5F8D and also suggests a therapeutic approach to anticoagulation via targeting of MCFD2 in complex with ERGIC-53. Moreover, our findings offer a potential approach for improvement in the production of recombinant glycoproteins of biopharmaceutical interest, simply by tagging them with a 10-amino-acid sequence. Because validity of the passport sequence is restricted depending on its position and spatial arrangement with respect to the N-glycans displayed on cargos, appropriate molecular designs would be required to maximize benefits from this approach in biopharmaceutical applications.

## Methods

### Preparation of proteins

The preparation of ERGIC-53^CRD^, MCFD2, and their complexes was performed as previously described^16,18^. FVIII fragment (Asn776–Asp838) was prepared using the protocol described previously^25^. The DNA fragment encoding residues Asn776–Asp838 of FVIII was cloned into the ubiquitin (Ub)-fused pET28a plasmid^25^. The peptide fragment was expressed as a Ub-fused protein in Luria–Bertani broth or M9 minimal medium supplemented with [^15^N]NH_4_Cl and/or [^13^C]glucose for NMR experiments. The Ub-fused FVIII peptide was purified with immobilized Ni^2+^-affinity chromatography and then digested with Ub carboxyl-terminal hydrolase (YUH1). The resultant peptide was purified by reversed-phase chromatography.

### NMR spectroscopy

NMR measurements were performed using AVANCE 800 and AVANCE 500 spectrometers each equipped with a 5-mm triple-resonance cryogenic probe (Bruker BioSpin, Fallanden, Switzerland). The NMR data were processed and analyzed using TopSpin (Bruker BioSpin) and SPARKY software^26^.

The chemical shifts of the FVIII fragment (Asn776–Asp838) were assigned by a standard protocol^18,25^. To obtain the dissociation constant based on NMR peak attenuation, 0.1 mM [^15^N]FVIII fragment was titrated with 0.02–0.3 mM of MCFD2/ERGIC-53^CRD^ or MCFD2 only in 20 mM 2-(*N*-morpholino) ethanesulfonic acid (MES; pH 6.0) containing 10 mM CaCl_2_, 150 mM NaCl, and 10% (v/v) D_2_O. Regarding [^15^N]MCFD2, NMR experiments were performed as described previously^18^; two molar excess amounts of peptides corresponding to Asp926–Lys953 of FV and Asn776–Asp838 of FVIII were individually added to 0.2 mM [^15^N]MCFD2. These experiments were performed in the presence or absence of five molar excess amounts of a tridecapeptide derived from Arg44–His56 of ERGIC-53 or that from Arg31–Arg44 of ERGL.

### Generation of KO cells using CRISPR/Cas9 genome editing

The guide RNAs (gRNAs) flanking the target enhancer regions for ERGIC-53 and MCFD2 were designed using ToolGen’s dRGEN synthesis service (Seoul, South Korea). The target DNA sequences of the gRNAs used in the present study were as follows: for MCFD2 deletion, CAGGCCCATGCTGCCGGGTTGGG, and for ERGIC-53 deletion, TTGTACTCGAAACGGCGATGTGG. The gRNA expression plasmids (pRGEN-U6-sgRNA) were purchased from ToolGen. To generate the KO cells of MCFD2 or ERGIC-53, individual gRNA plasmids and a plasmid-expressing Cas9-green fluorescent protein (GFP) (Addgene, catalog #44719) were transiently transfected into HCT116 cells, which are diploid cells suitable for genome editing using the CRISPR/Cas9 method^27^, in a six-well plate using polyethylenimine “Max” (Polysciences Inc., Warrington, PA) and a Cas9:gRNA molar ratio of 1:5 as described previously^28^. At 48 h post transfection, cells with high GFP expression were identified using fluorescence-activated cell sorting with the BD FACSAria II cell sorter (BD Biosciences, San Jose, CA). For knocking out ERGL in the wild-type and ERGIC-53-KO cells, the Guide-it CRISPR/Cas9 System (green/red) (Takara Bio Inc., Kusatsu, Japan) was used for the cloning and expression of target single gRNAs, whose target ERGL DNA sequences were AGCTCAFCTTCAAAGGCCCA and TTGTACTCAAACCTGCGTAG. The two constructed pGuide-it Vectors (green and red) were transiently transfected into HCT116 cells. At 48 h post transfection, cells with high ZsGreen1 and tdTomato expression were identified using fluorescence-activated cell sorting. Sorted cells were plated into individual wells of a 96-well plate and then re-plated as single cells in 6 cm dishes. The deletions in the clones were confirmed by polymerase chain reaction and sequencing analysis (Supplementary Figs. 8, 9, 10, and 11).

### Secretion level assays of recombinant proteins

For expression of the FVIII and FV full-length and deletion mutant proteins in mammalian cells, the codon-optimized FVIII and FV genes were inserted into the p3XFLAG-CMV-14 Expression Vector and p3XFLAG-CMV-9 Expression Vector, respectively (Sigma-Aldrich, St. Louis, MO). For the expression of the wild-type and mutated AAT, EPO, and sFγRIIIa in mammalian cells, the corresponding genes (purchased from FASMAC Co., Ltd., Atsuki, Japan) were inserted into the p3XFLAG-CMV-9 Expression Vector (Sigma-Aldrich).

HCT116 and its mutated cells as well as HEK293T were maintained in Dulbecco’s modified Eagle’s medium (DMEM) supplemented with 10% fetal bovine serum in 5% CO_2_ at 37 °C. For complementary DNA (cDNA) transfection, cells were grown overnight and transfected using Polyethylenimine “Max” (Polysciences Inc.) as described previously^28^. Expi293F cells (Thermo Fisher Scientific, Waltham, MA) were maintained in Expi293 Expression Medium (Thermo Fisher Scientific) in 5% CO_2_ at 37 °C on an orbital shaker platform rotating at 125 r.p.m. For cDNA transfection, cells were transfected with the ExpiFectamine 293 Transfection Kit (Thermo Fisher Scientific Inc.). Protein expression was performed in serum-free medium.

The secreted levels of FVIII and FV antigens in the culture medium were determined with an sandwich enzyme-linked immunosorbent assay (ELISA) method using the VisuLize FVIII Antigen Kit (Affinity Biologicals Inc., Ancaster, ON) and the Asssay Max™ Human Factor V ELISA Kit (ASSAYPRO, MO), respectively. The secreted levels of AAT and EPO antigens in the culture medium were determined with an ELASA method using the anti-AAT antibody (Agilent, Santa Clara, CA) and anti-Flag M2 antibody (Sigma-Aldrich), respectively.

### Western blotting

Western blots were performed according to methods in previous studies^29,30^. At 72 h post transfection, the EPO and sFγRIII recombinant proteins were purified with anti-Flag M2 Affinity Gel (Sigma-Aldrich). The gel was washed twice with phosphate-buffered saline, and proteins were eluted with the 3× FLAG tag peptide (Sigma-Aldrich). The purified proteins were subjected to 3–10% gradient sodium dodecyl sulfate-polyacrylamide gel electrophoresis and subsequently transferred to a polyvinylidene difluoride membrane (Millipore, Billerica, MA). After blocking with Blocking One solution (Nacalai Tesque, Kyoto, Japan), the membranes were incubated with monoclonal mouse anti-Flag M2 (Sigma-Aldrich, F1804), anti-human LMAN1 (ERGIC-53) (Proteintech, Tokyo, Japan, 13364), anti-human SDNSF/MCFD2 (R&D System, Minneapolis, MN, MAB2357), and anti-β-actin (Sigma-Aldrich, A2228) antibodies, and then with horseradish peroxidase (HRP)-conjugated secondary antibody [anti-mouse IgG antibody-HRP, GE Healthcare (Piscataway, NJ) and anti-rabbit Ig antibody-HRP, Cell Signaling Technology (Danvers, MA)] in 20 mM Tris-HCl (pH 7.6) containing 150 mM NaCl and 0.05% Tween-20. The protein bands were visualized with Immobilon Western Chemiluminescent HRP substrate solution (Millipore).

### Reverse transcription-PCR analysis

Total RNAs were isolated from HCT116 using TRIzol reagent (Thermo Fisher Scientific) and used as templates for synthesizing cDNAs by SuperScriptIII reverse transcriptase (Thermo Fisher Scientific). PCR was performed using AmpliTaq Gold DNA polymerase (Thermo Fisher Scientific) with the primer sequences as follows (5′–3′): AAAAGCGGCCTTTGAGAACTGG and TGGTCATCTGCAAGACCTCCAG for ERGIC-53; CCCAGGCTGAGAGACAATGGAA and GCCGAAGAAGCCTACAGTCTGA for ERGL. The PCR products were analyzed by agarose gel electrophoresis using 2% agarose gels containing SYBR safe DNA Gel stain (Thermo Fisher Scientific).

### Retention using selective hooks system

For live-cell imaging using RUSH system^24^, li-Str_ST-SBP-mCherry (Plasmid #65269) was purchased from Addgene. We replaced mCherry with mScarlet-I-EPO or mScarlet-I-EPO-motif (*Sbf*I/*Xba*I). HCT116 cells were plated on 35-mm glass-based dishes, which were coated with poly-l-lysine, and grown overnight. RUSH plasmids to express mScarlet-I-EPO and mScarlet-I-EPO-motif were individually transfected into the cells using Expifectamine^TM^ 293 reagent. After 24 h, the medium was changed into DMEM including 40 μM (+)-biotin for starting the translocation form ER. After 30 min incubation for the translocation, cells were then fixed with 2% paraformaldehyde for 10 min at room temperature. After blocking with Blocking One solution (Nacalai Tesque), the cells were incubated with polyclonal rabbit anti-GM130 (Proteintech, 11308-1-AP, dilution 1 :1000) and then with Alexa Fluor 488-conjugated anti-rabbit IgG antibody [Jackson ImmunoResearch (West Grove, PA), 711-545-152, dilution 1:1000] in 20 mM Tris-HCl (pH 7.6) containing 150 mM NaCl and 0.05% Tween-20. Cell images were recorded using a fluorescence microscope BZ-X700 and BZ-H3XF/Sectioning Module (Keyence Co., Osaka, Japan) and analyzed by Fiji^31^. For quantification of co-localization of EPO proteins and GM130, the images were thresholded by the Otsu method and analyzed by calculating Manders’ coefficient (*M*1) with Coloc2 plugin^32^. Because of abnormal distribution of the imaging data based on Shapiro–Wilk normality test, nonparametric statistics were used for the analysis. All statistical analysis was performed in the R statistical environment version 3.5.3^33^.

### Reporting summary

Further information on research design is available in the Nature Research Reporting Summary linked to this article.

## Supplementary information

Supplementary Information

Reviewers comments

Reporting Summary

## Data Availability

NMR chemical shift assignments of MCFD2 and the FVIII peptide are available in the Biological Magnetic Resonance Data Bank (BMRB) under entry ID 12043 [http://www.bmrb.wisc.edu/data_library/summary/index.php?bmrbId=12043] and 12044 [http://www.bmrb.wisc.edu/data_library/summary/index.php?bmrbId=12044], respectively. The datasets of the current study are available from the corresponding authors on reasonable request. The source data underlying Figs. 1c, 2b, 3b, 4b–f, and 5b and Supplementary Figs. 2A, 3C, 4, 5, 6C, 7, 8C, and 9C are provided as a Source Data file.

## Source Data

Source Data
