# Supplementary material for: Improved secretion of glycoproteins using an N-glycan-restricted passport sequence tag recognized by cargo receptor
Source: Nat Commun. 2020 Mar 13;11:1368. doi: 10.1038/s41467-020-15192-1 (PMC7069976; doi:10.1038/s41467-020-15192-1)
Supplement: Supplementary file 1 — Supplementary Information [file 41467_2020_15192_MOESM1_ESM.pdf]

Supplementary Information

Improved secretion of glycoproteins using an N-glycan-restricted passport sequence tag recognized by cargo receptor

Hirokazu Yagi[1], Maho Yagi-Utsumi[1,2,3,4], Rena Honda[1,3,4], Yusaku Ohta[2,5], Taiki Saito[1], Miho Nishio[1], Satoshi Ninagawa[3], Kousuke Suzuki[1], Takahiro Anzai[3], Yukiko Kamiya[3], Kazuhiro Aoki[2,5], Mahito Nakanishi[6], Tadashi Satoh[1], and Koichi Kato[1,2,3,4,*]

[1] Graduate School of Pharmaceutical Sciences, Nagoya City University, 3-1 Tanabe-dori, Mizuho-ku, Nagoya 467-8603, Japan

[2] Exploratory Research Center on Life and Living Systems (ExCELLS), National Institutes of Natural Sciences, 5-1 Higashiyama, Myodaiji-cho, Okazaki, 444-8787, Japan

[3] Institute for Molecular Science, National Institutes of Natural Sciences, 5-1 Higashiyama, Myodaiji, Okazaki, Aichi 444-8787, Japan

[4] School of Physical Science, SOKENDAI (The Graduate University for Advanced Studies), 5-1 Higashiyama, Myodaiji, Okazaki, 444-8787, Japan.

[5] National Institute for Basic Biology, National Institutes of Natural Sciences, 5-1 Higashiyama, Myodaiji, Okazaki, Aichi 444-8787, Japan.

[6] Biotechnology Research Institute for Drug Discovery, National Institute of Advanced Industrial Science and Technology (AIST), 1-1-1, Higashi, Central 5 Tsukuba, Ibaraki, 305-8565 Japan.

[*]Addresses for correspondence: Koichi Kato, Ph.D., Exploratory Research Center on Life and Living Systems and Institute for Molecular Science, National Institutes of Natural Sciences, 5-1 Higashiyama, Myodaiji, Okazaki, Aichi 444-8787, Japan, Tel. +81-564-59-5225, Fax: +81-564-59-5224, e-mail: kkatonmr@ims.ac.jp

Supplementary Figures

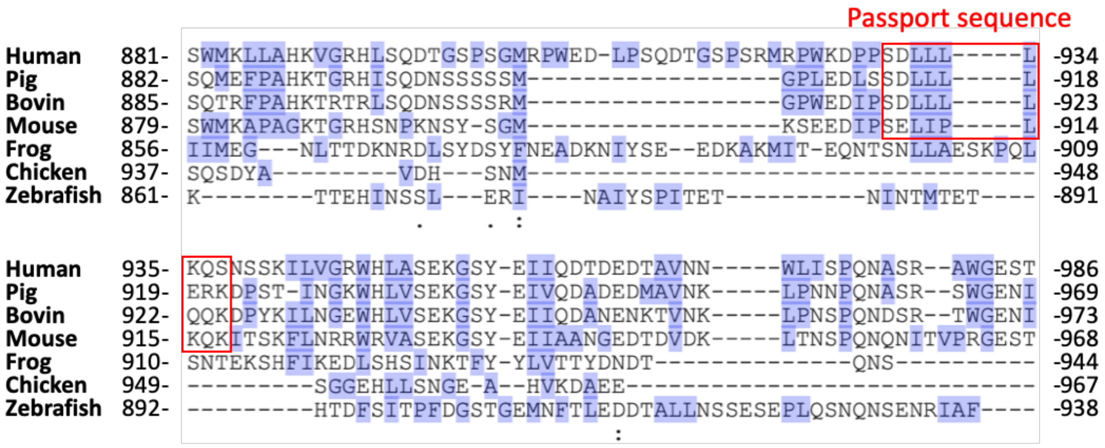

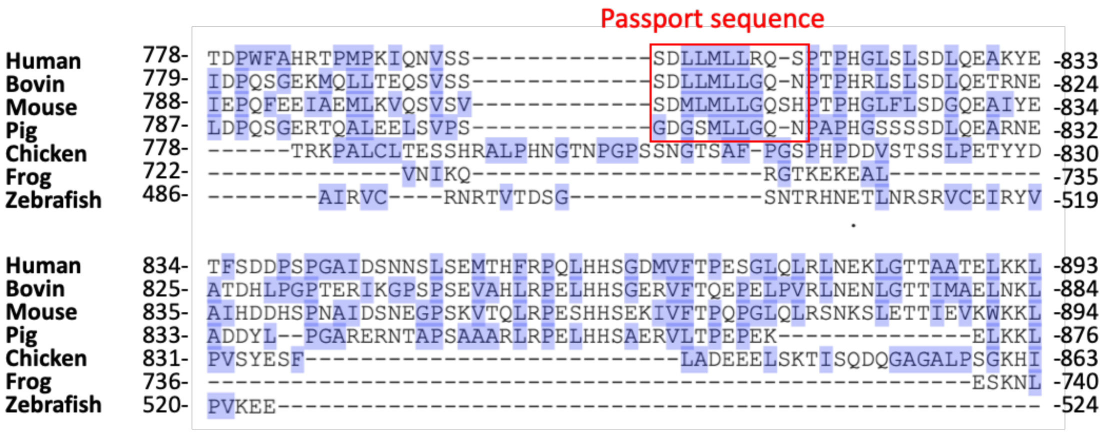

Supplementary Fig. 1. Multiple alignments of the MCFD2-binding motif sequences on FV and FVIII sequences. The FV and FVIII sequences were aligned with sequences from TrEMBL databases using ClustalW. Hydrophobic residues are shaded in blue. The MCFD2-binding motif sequences are boxed in red. The amino acid sequences annotated in the UniProt database (P12259, Q9GLP1, Q28107, Q88783, K9J7M8, A0A1D5P5L2, Q90X47, P00451,G5E5W1, Q06194, P12263, F1NPT2, A0A1L8F225, and A0A0R4INL6) are presented.

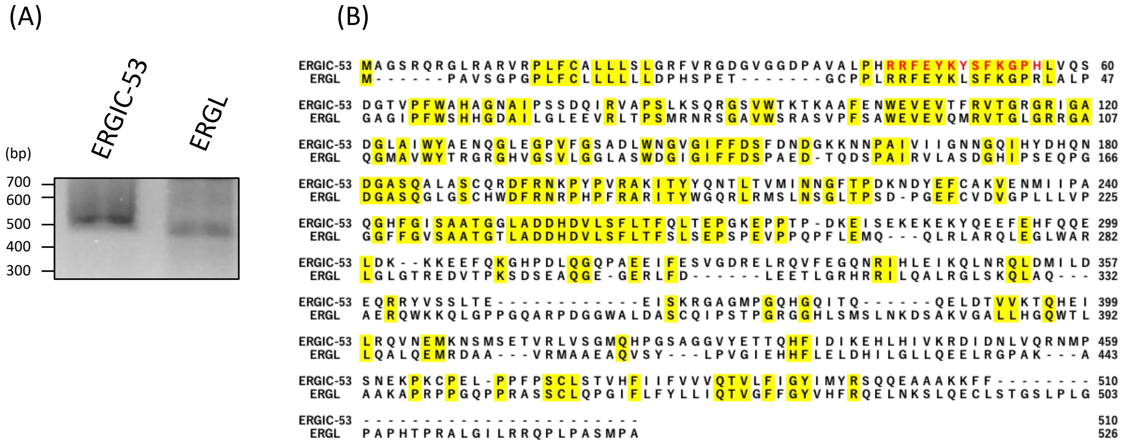

Supplementary Fig. 2. ERGIC-53-like protein, ERGL. (A) ERGL was expressed in HCT116 cells. The mRNA expression of ERGIC-53 and ERGL in HCT116 was analyzed by RT-PCR. Consistent data were obtained from three independent experiments. (B) Sequence alignments of ERGIC−53 and ERGL. Sequences were aligned using Clustal Omega (https://www.ebi.ac.uk/Tools/msa/clustalo/). Conserved residues are shaded in yellow. The residues involved in the MCFD2-binding site of ERGIC-53 are shown in red. The amino acid sequences annotated in the UniProt database (P49257 and Q9HAT1) are presented.

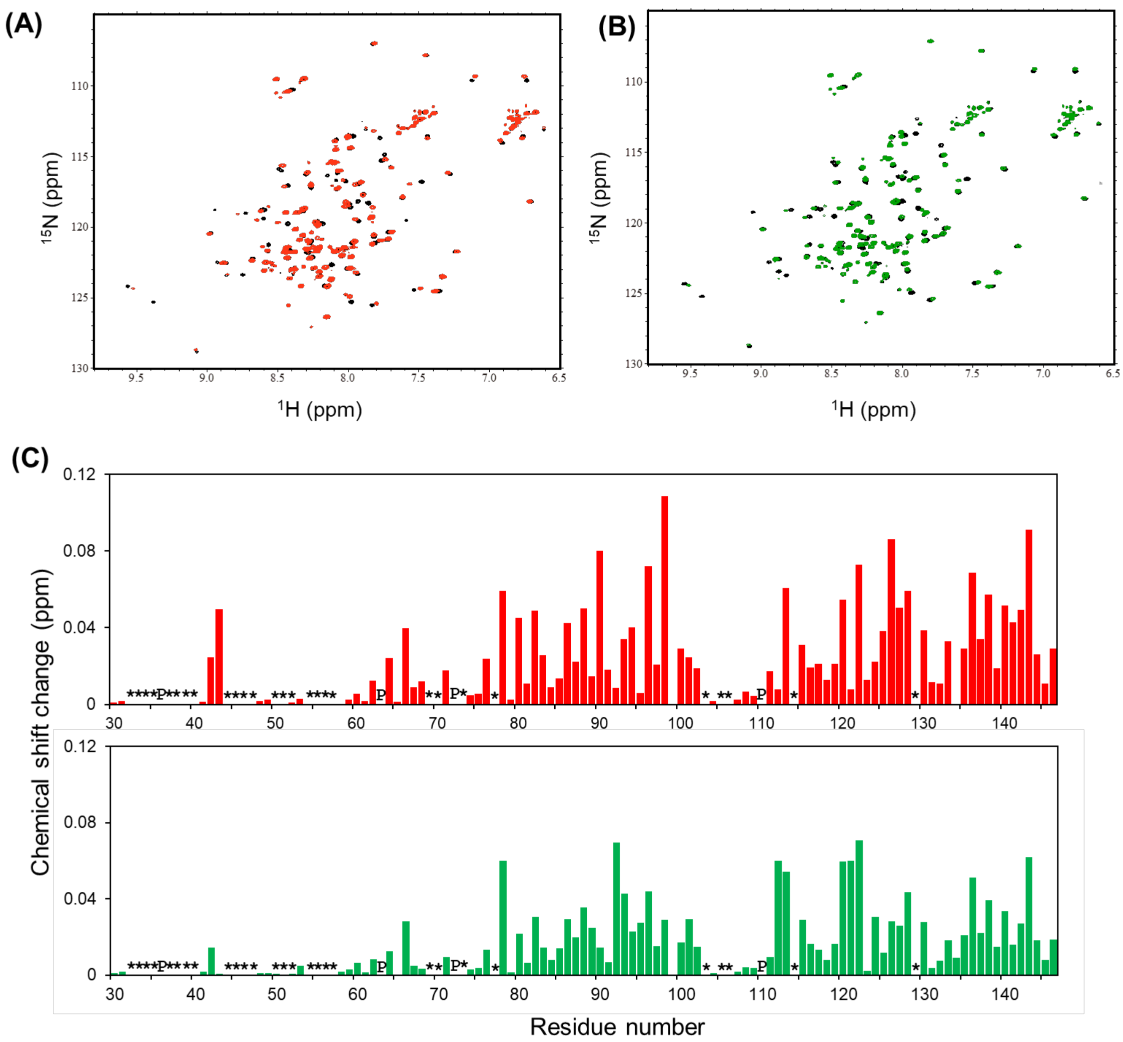

Supplementary Fig. 3. NMR characterization of the interaction of MCFD2 and FV-derived peptide depending on the ERGL-derived peptide. (A) ^{1}H–^{15}N HSQC spectra of [^{15}N]MCFD2 with five molar equivalents of the ERGL-derived peptide (RRFEYKLSFKGPR) in the presence (red) and absence (black) of two molar equivalents of the FV peptide (DPPSDLLLLKQSNSSKILVGRWHLASEK) (left). (B) ^{1}H–^{15}N HSQC spectra of [^{15}N]MCFD2 in the presence (green) and absence (black) of two molar equivalents of the FV peptide. The synthetic FV-derived peptide was purchased from FASMAC, and dissolved in 100% dimethyl sulfoxide-d6 (DMSO-d6). Proteins were dissolved in 20 mM MES (pH 6.0) containing 10 mM $CaCl_2$, 150 mM NaCl, 2% (v/v) DMSO-d6, and 10% (v/v) D_2O. NMR spectra were acquired at 303 K. (C) NMR chemical shift changes observed for MCFD2 in the presence (upper) and absence (lower) of the ERGL-derived peptide upon addition of the FV-derived peptide. In the NMR perturbation profiles, definition of asterisks, “P”, and chemical shift changes are the same as those in Figure 2.

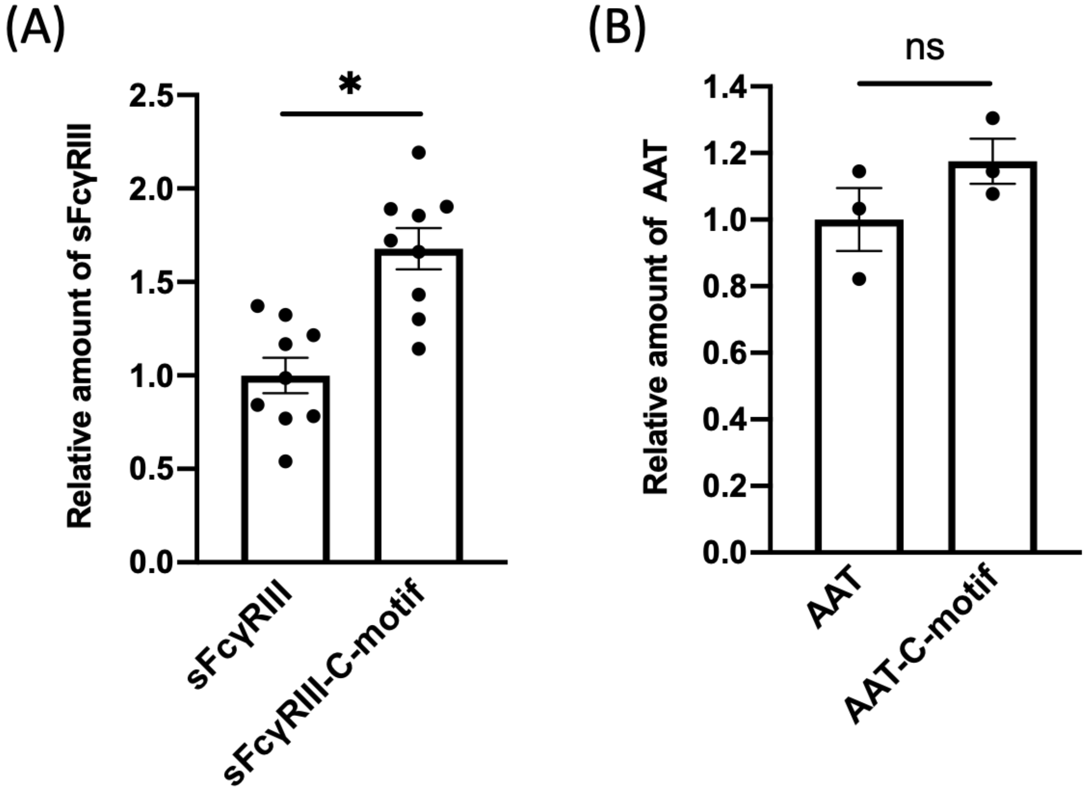

Supplementary Fig. 4. Tagging with the SDLLMLLRQS motif caused increased secretion of sFγRIII and α1-antitrypsin (AAT) from HCT116 cells. (A) The secretion level of sFγRIII with or without the C-terminal SDLLMLLRQS motif (sFγRIII-C-motif) was measured in the culture medium using an enzyme-linked immunosorbent assay (ELISA). Error bars represent the standard error of the mean (S.E.M.) (n = 9 independent transfections). Significant differences were calculated compared with control sFγRIII expression levels using two-tailed unpaired Student's t-test (* $p < 0.01$). **(B)** The secretion level of AAT with or without the C-terminal SDLLMLLRQS motif (AAT-motif) was measured in the culture medium using the ELISA. Error bars represent the standard error of the mean (S.E.M.) (n =3 independent transfections). Significant differences were calculated compared with control AAT expression levels using two-tailed unpaired Student's t-test (ns p >0.05).

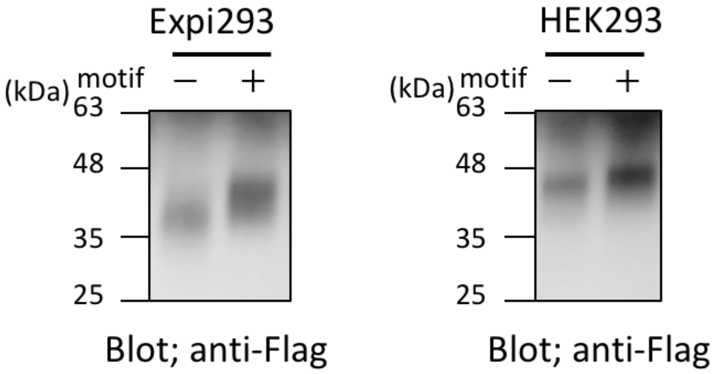

Supplementary Fig. 5. Tagging with the SDLLMLLRQS motif caused increased secretion of EPO from Expi293 and HEK293 cells. Expression levels of EPO proteins with or without the C-terminal SDLLMLLRQS motif were analyzed by Western blotting with anti-Flag antibody. Consistent data were obtained from two independent experiments.

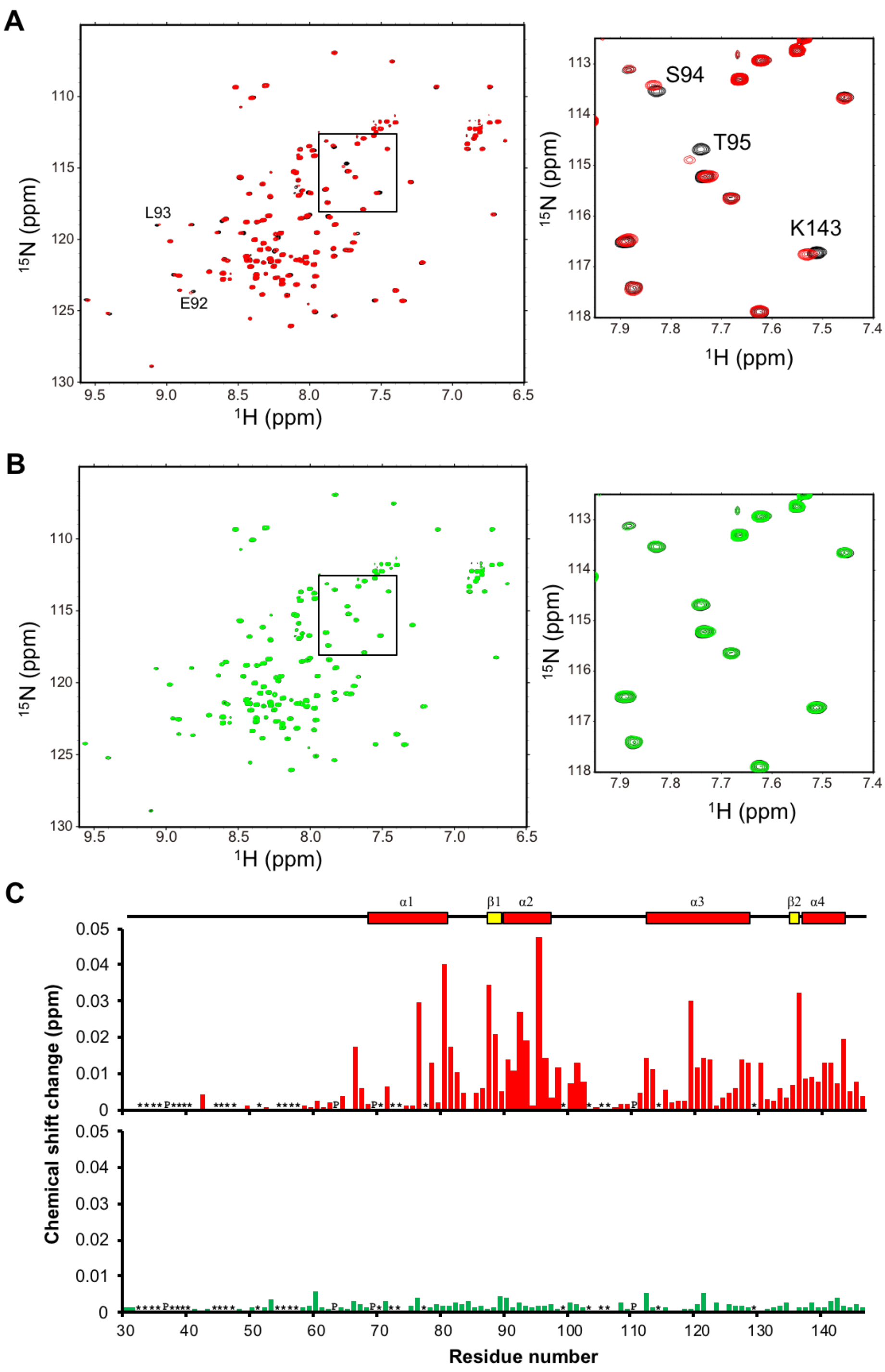

Supplementary Fig. 6. Effect of amino acid substitution in the passport sequence on its interaction with MCFD2. (A, B) ^{1}H–^{15}N HSQC spectra of [^{15}N]MCFD2 in the absence (black) or presence of (A) the FVIII-derived peptide (SDLLMLLRQS, red) or (B) its alanine-substituted peptide (SDAAAAARQS, green) at an equivalent molar ratio. The close views of spectral regions (boxed) are displayed at the right. These experiments were performed in the presence of fivefold

molar excess of the ERGIC-53^{CRD}-derived peptide. The synthetic FVIII-derived peptide and its alanine-substituted peptide were purchased from Toray Research Center, Inc., and dissolved in 100% DMSO-d6. Proteins were dissolved in 20 mM MES (pH 6.0) containing 10 mM $CaCl_2$, 150 mM NaCl, 9.75% (v/v) dimethyl sulfoxide, 0.25% (v/v) DMSO-d6, and 10% (v/v) D_2O. NMR spectra were acquired at 303 K. (C) NMR chemical shift changes observed for MCFD2 in the presence of the ERGIC-53^{CRD}-derived peptide upon addition of the FVIII-derived peptide (upper) or its alanine-substituted peptide (lower). In the NMR perturbation profiles, definitions of asterisks, “P”, and chemical shift change are the same as those in Figure 2.

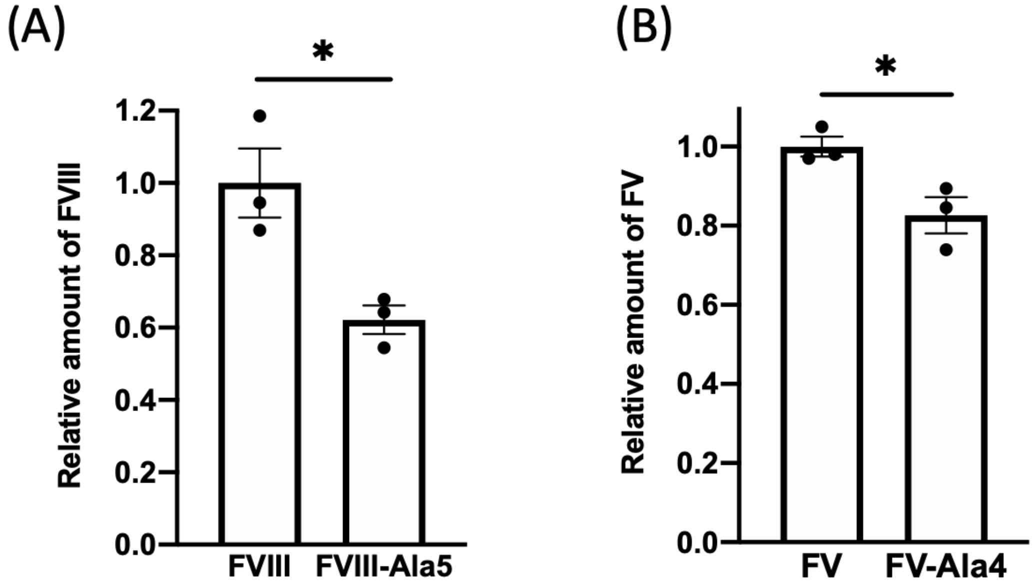

Supplementary Fig. 7. The replacement of the leucine cluster of FVIII and FV resulted in significant reduction in the secretion level. (A) Replacement of the LLMLL motif with AAAAA caused a reduction in the secretion of FVIII from HCT116 cells. Wild-type FVIII and its motif-substituted mutant (FVIII-Ala5) were expressed in HCT116 cells. (B) Replacement of the LLLL motif with AAAA caused a reduction in the secretion of FV from COS-7 cells. Wild-type FV and its motif-substituted mutant (FV-Ala4) were expressed in COS-7 cells. The secretion levels of FVIII and FV antigens in the culture medium were determined using a commercial sandwich ELISA. Error bars represent the S.E.M. (n = 3 independent transfections). Significant differences were calculated compared with the wild-type FVIII or wild-type FV expression levels using two-tailed unpaired Student’s t-test (* $p < 0.05$).

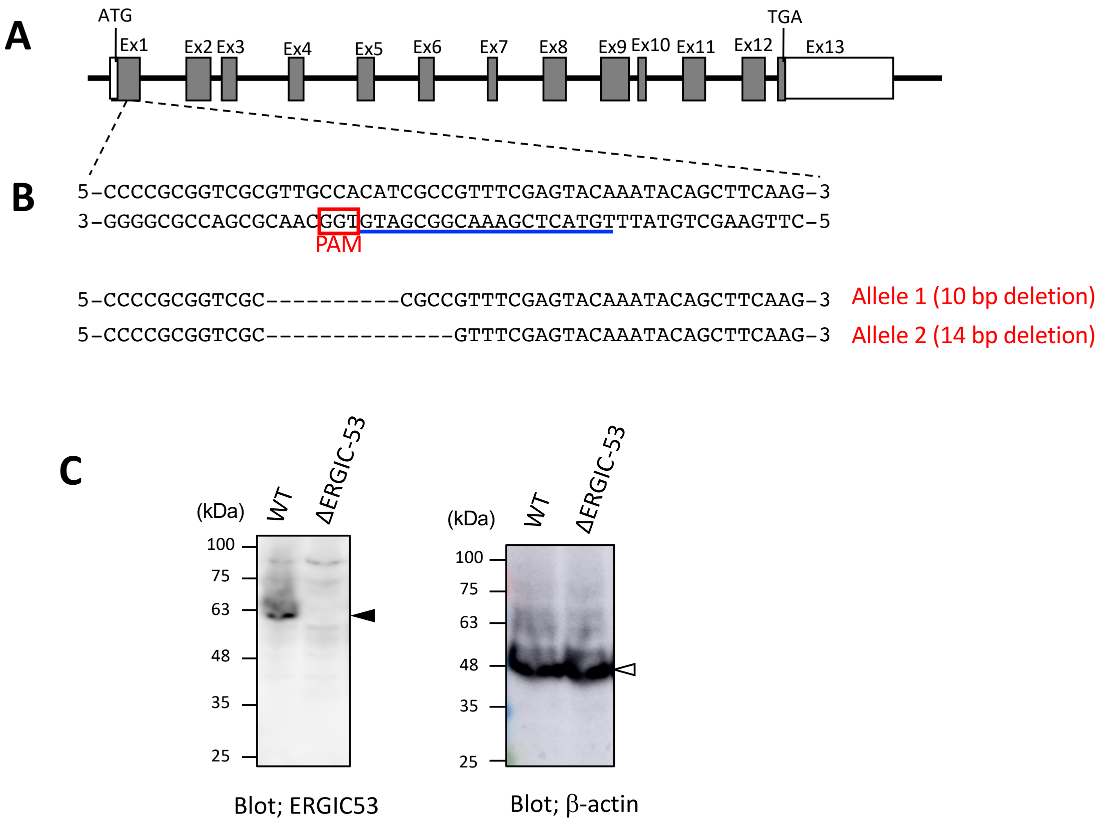

Supplementary Fig. 8. Editing of *LMAN1* gene by the CRISPR/Cas9 system. (A) Schematic representation of the genomic target sites in the *LMAN1* gene. Exons, indicated by rectangles, are numbered from Ex1 to Ex13. The coding portions of the gene are shaded. The open portions of exons 1 and 13 represent the 5′ UTR and 3′ UTR, respectively. (B) Genotyping results of ERGIC-53 KO cell. Sequence analysis confirmed *LMAN1* mutations, with 10- and 14-ucleotide deletions in allele 1 and allele 2, respectively. Blue line shows the sequence of gRNAs; protospacer adjacent motifs (PAM) are labeled in red; -, nucleotide deletion. (C) ERGIC-53 protein expression level in wild-type (WT) and ERGIC-53 KO cells (ΔERGIC-53) were analyzed by Western blotting with anti-ERGIC-53 (black arrow) and anti-β-actin antibodies (white arrow). Consistent data were obtained from two independent experiments.

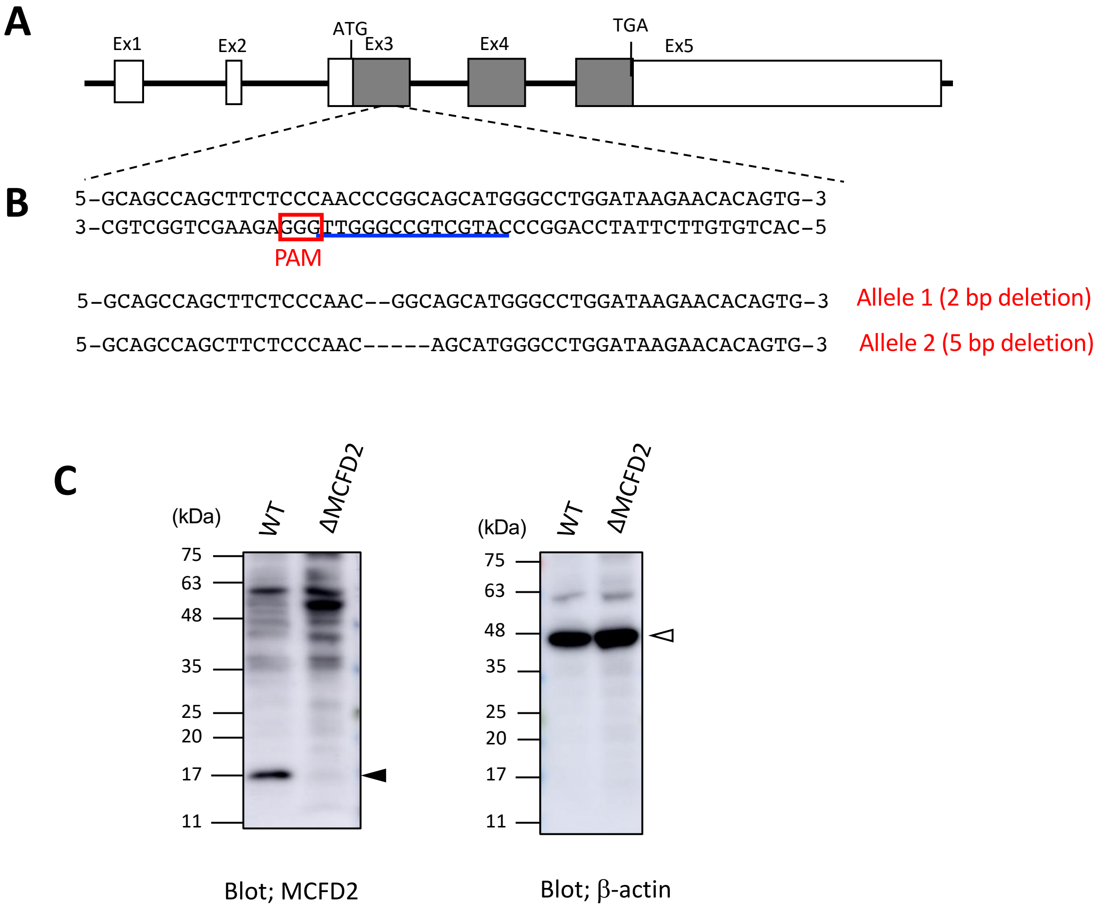

Supplementary Fig. 9. Editing at *MCFD2* gene by the CRISPR/Cas9 system. (A) Schematic representation of the genomic target sites in the *MCFD2* gene. Exons, indicated by rectangles, are numbered from Ex1 to Ex5. The coding portions of the gene are shaded. The open portions of exons 3 and 5 represent the 5′ UTR and 3′ UTR, respectively. (B) Genotyping results of MCFD2 KO cell. Sequence analysis confirmed the *MCFD2* mutations, with 2- and 5-nucleotide deletions in allele 1 and allele 2, respectively. Blue line shows the sequence of gRNAs; protospacer adjacent motifs (PAM) are labeled in red; -, nucleotide deletion. (C) MCFD2 protein expression level in wild-type (WT) and MCFD2 KO cells (ΔMCFD2) were analyzed by Western blotting with anti-MCFD2 (black arrow) and anti-β-actin antibodies (white arrow). Consistent data were obtained from two independent experiments.

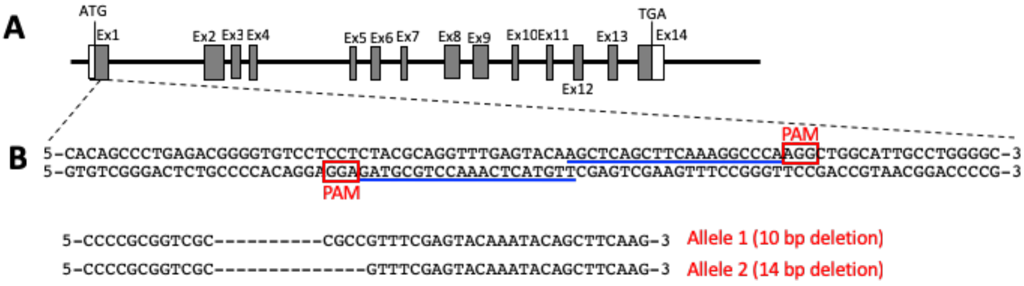

Supplementary Fig. 10. Editing at *LMAN1L* gene in wild-type HCT116 cells by the CRISPR/Cas9 system. (A) Schematic representation of the genomic target sites in the *LMAN1L (lectin, mannose binding 1 like)* gene. Exons, indicated by rectangles, are numbered from Ex1 to Ex14. The coding portions of the gene are shaded. The open portions of exons 1 and 14 represent the 5′ UTR and 3′ UTR, respectively. (B) Genotyping results of ERGL KO cell. Sequence analysis confirmed the *LMAN1L* mutations, with 10- and 14-nucleotide deletions in allele 1 and allele 2, respectively. Blue line shows the sequence of gRNAs; protospacer adjacent motif (PAM) is labeled in red; -, nucleotide deletion.

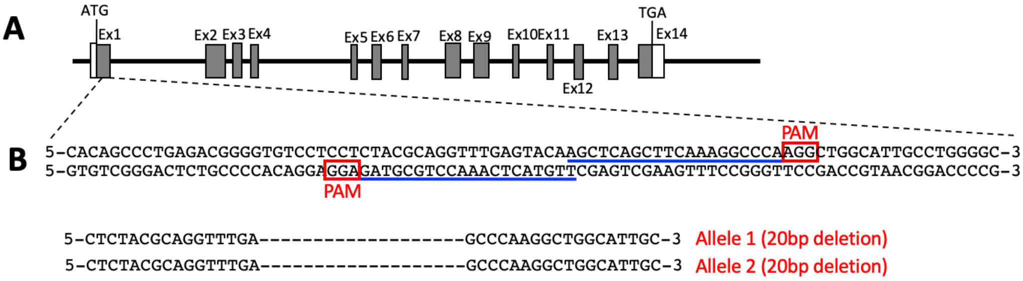

Supplementary Fig. 11. Editing at *LMAN1L* gene in ERGIC-53-KO cells by the CRISPR/Cas9 system. Editing at *LMAN1L* gene by the CRISPR/Cas9 system. (A) Schematic representation of the genomic target sites in the *LMAN1L* gene. Exons, indicated by rectangles, are numbered from Ex1 to Ex14. The coding portions of the gene are shaded. The open portions of exons 1 and 14 represent the 5′ UTR and 3′ UTR, respectively. (B) Genotyping results of ERGL KO cell. Sequence analysis confirmed the *LMAN1L* mutations, with 20- and 20-nucleotide deletions in allele 1 and allele 2, respectively. Blue line shows the sequence of gRNAs; protospacer adjacent motif (PAM) is labeled in red; -, nucleotide deletion.
