# Supplementary material for: Improved secretion of glycoproteins using an N-glycan-restricted passport sequence tag recognized by cargo receptor
Source: Nat Commun. 2020 Mar 13;11:1368. doi: 10.1038/s41467-020-15192-1 (PMC7069976; doi:10.1038/s41467-020-15192-1)
Supplement: Supplementary file 3 — Reporting Summary [file 41467_2020_15192_MOESM3_ESM.pdf]

nature research

Corresponding author(s): Koichi Kato

Last updated by author(s): Feb 11, 2020

Reporting Summary

Nature Research wishes to improve the reproducibility of the work that we publish. This form provides structure for consistency and transparency in reporting. For further information on Nature Research policies, see Authors & Referees and the Editorial Policy Checklist .

Statistics

For all statistical analyses, confirm that the following items are present in the figure legend, table legend, main text, or Methods section.

n/a	Confirmed	
☐	☒	The exact sample size (*n*) for each experimental group/condition, given as a discrete number and unit of measurement
☐	☒	A statement on whether measurements were taken from distinct samples or whether the same sample was measured repeatedly
☐	☒	The statistical test(s) used AND whether they are one- or two-sided<br>*Only common tests should be described solely by name; describe more complex techniques in the Methods section.*
☒	☐	A description of all covariates tested
☐	☒	A description of any assumptions or corrections, such as tests of normality and adjustment for multiple comparisons
☐	☒	A full description of the statistical parameters including central tendency (e.g. means) or other basic estimates (e.g. regression coefficient) AND variation (e.g. standard deviation) or associated estimates of uncertainty (e.g. confidence intervals)
☐	☒	For null hypothesis testing, the test statistic (e.g. *F*, *t*, *r*) with confidence intervals, effect sizes, degrees of freedom and *P* value noted<br>*Give P values as exact values whenever suitable.*
☒	☐	For Bayesian analysis, information on the choice of priors and Markov chain Monte Carlo settings
☒	☐	For hierarchical and complex designs, identification of the appropriate level for tests and full reporting of outcomes
☒	☐	Estimates of effect sizes (e.g. Cohen's *d*, Pearson's *r*), indicating how they were calculated

Our web collection on statistics for biologists contains articles on many of the points above.

Software and code

Policy information about availability of computer code

Data collection	No software was used for data collection in this study.
Data analysis	The NMR data were processed and analyzed using TopSpin 3.5.7 and SPARKY 3.114; Protein models were visualized using PyMOL 2.0; Fuji including ImageJ 1.48v was used to analyze density of the band on SDS-PAGE and protein localization of fluorescence images; All statistical analysis was performed in Prism 8 and R statistical environment version 3.5.3.

For manuscripts utilizing custom algorithms or software that are central to the research but not yet described in published literature, software must be made available to editors/reviewers. We strongly encourage code deposition in a community repository (e.g. GitHub). See the Nature Research guidelines for submitting code & software for further information.

Data

Policy information about availability of data

All manuscripts must include a data availability statement. This statement should provide the following information, where applicable:

- Accession codes, unique identifiers, or web links for publicly available datasets
- A list of figures that have associated raw data
- A description of any restrictions on data availability

PDB code: 1CFF has been referenced in the manuscript. UniProt database: P49257, Q9HAT1, P12259, Q9GLP1, Q28107, Q88783, K9J7M8, A0A1D5P5L2, Q90X47, P00451,G5E5W1, Q06194, P12263, F1NPT2, A0A1L8F225, and A0A0R4INL6 have been referenced as amino acid sequences in the manuscript.
The authors declare that all data supporting the findings of this study are available within the paper and its supplementary information. Raw data files are available from the corresponding author upon reasonable request.

Field-specific reporting

Please select the one below that is the best fit for your research. If you are not sure, read the appropriate sections before making your selection.

- [x] Life sciences
- [ ] Behavioural & social sciences
- [ ] Ecological, evolutionary & environmental sciences

For a reference copy of the document with all sections, see nature.com/documents/nr-reporting-summary-flat.pdf

Life sciences study design

All studies must disclose on these points even when the disclosure is negative.

Sample size	Sample sizes were similar as reported in previous publications [Blood (2004) 103, 3412-3419 and Blood (2010) 116, 640-648].
Data exclusions	No data exclusions were taken for this manuscript.
Replication	Each cell-based experiment was repeated at least three times as described in Figure legends. Experimental findings were reliably reproduced between these experiments.
Randomization	No experiments presented in this study required randomization.
Blinding	No experiments presented in this study required blinding.

Reporting for specific materials, systems and methods

We require information from authors about some types of materials, experimental systems and methods used in many studies. Here, indicate whether each material, system or method listed is relevant to your study. If you are not sure if a list item applies to your research, read the appropriate section before selecting a response.

Materials & experimental systems

n/a	Involved in the study	
☐	☒	Antibodies
☐	☒	Eukaryotic cell lines
☒	☐	Palaeontology
☒	☐	Animals and other organisms
☒	☐	Human research participants
☒	☐	Clinical data

Methods

n/a	Involved in the study	
☒	☐	ChIP-seq
☒	☐	Flow cytometry
☒	☐	MRI-based neuroimaging

Antibodies

Antibodies used	Antibodies used for immunoblotting;<br>mouse anti-FLAG antibody, M2 clone (Sigma-Aldrich, F1804, 1:5000), rabbit anti-ERGIC-53 polyclonal antibody (Proteintech, 13364, 1:1000), mouse anti-human SDNSF/MCFD2 monoclonal antibody, Clone # 294301 (R&D System, MAB2357, 1:1000), rabbit anti-β-actin monoclonal antibody, AC-74 clone (Sigma-Aldrich, A2228, 1:2500), and anti-mouse IgG antibody-HRP (GE healthcare, NA931, 1:3000)<br><br>Antibodies used for cytoimmunostaining;<br>rabbit anti-GM130 polyclonal antibody (Proteintech, 11308-1-AP, 1:250) and Alexa Fluor 488-conjugated anti-rabbit IgG antibody (Jackson ImmunoResearch, 711-545-152, 1:250).
Validation	Validation details are available from the manufacture for each commercial antibody used in this study. In addition, mouse anti-FLAG antibody and anti-β-actin monoclonal antibody are validated in the previous report [Scientific Reports (2013) 3, 3288].

Eukaryotic cell lines

Policy information about cell lines

Cell line source(s)	HCT116 cells were purchased from ATCC. Expi293T cells used in this study were obtained from Thermo Fisher Scientific. The cell lines on this study were generated for this study, including ERGIC-53, MCFD2, and ERGL deficient HCT116 cells.
Authentication	The cell lines were not authenticated.
Mycoplasma contamination	Cell lines used in this study tested negative for mycoplasma.

Commonly misidentified lines<br>(See ICLAC register)	No commonly misidentified cell lines were used.
