# Supplementary material for: Improved secretion of glycoproteins using an N-glycan-restricted passport sequence tag recognized by cargo receptor
Source: Nat Commun. 2020 Mar 13;11:1368. doi: 10.1038/s41467-020-15192-1 (PMC7069976; doi:10.1038/s41467-020-15192-1)
Supplement: Supplementary file 2 — Reviewers comments [file 41467_2020_15192_MOESM2_ESM.pdf]

Reviewers' comments:

Reviewer #1 (Remarks to the Author):

This manuscript reports the identification of a peptide sequence in coagulation factors V and VIII, which the authors propose serves as a “passport tag" to mark these proteins for efficient trafficking through the secretory pathway via interactions with the MCFD2/LMAN1 cargo receptor. They then go on to use this tag to boost the secretion of an unrelated protein, erythropoietin (EPO). Though the authors hypothesis is of potential interest, there are number of significant concerns as outlined below.

1) The authors focus on the N-terminus of the FVIII B-domain to identify their “passport” tag, citing studies from reference 20 that show that these sequences “influence the secretion level of FVIII.” However, in reference 20 (Miao et al), though a significant increase in FVIII secretion is observed with addition of the first 54 aa of the B-domain, no further increase is observed with the addition of the next 63 aa (containing the current manuscript’s “passport” sequence). How can the findings of the current manuscript be reconciled with these earlier studies, which seem to exclude a major effect of this putative “passport” sequence?

2) Is this putative “passport” sequence uniquely conserved across species within the otherwise poorly conserved B-domain? Is the “passport” sequence also present in other putative MCFD2/LMAN1 cargoes, including alpha-1-antitrypsin and cathepsins C and Z? If not, how are these latter proteins targeted to LMAN1/MCFD2?

3) The authors rely on EPO as the sole experimental protein in which to test their hypothetical “passport” sequence. How generalizable is the effect of this passport sequence? Have the authors tried any other “typical” secreted proteins?

4) Are the dissociation constants observed for the factor VIII derived peptide with LMAN1/MCFD2 physiologically relevant?

5) The authors show that deletion of the MCFD2–binding motif from factor VIIII decreases secretion from HCT116 cells (figure 4). Is the same effect seen with factor V? In figure 4E, the authors explore the effect of point mutations in the “passport” sequence on the efficiency of EPO secretion, localizing the most crucial function to the central leucine cluster. Is this conclusion supported by mutations of the same motifs within factor VIII and/or V?

6) the authors explain the smaller effect of ERGIC53 deletion on factor VIII secretion compared to MCFD2 by proposing overlap with the related protein ERGL. Does simultaneously deletion of both ERGL and ERGIC53 confirm this hypothesis?

7) In the experiments depicted in figure 4, were appropriate controls employed to normalize for variations in transfection efficiency?

8) The authors might consider Endo-H treatment of cell lysates to examine the relative ER retention of the recombinant proteins under study.

Reviewer #3 (Remarks to the Author):

The manuscript by Yagi et al investigates the trafficking of FVIII and its its regulation by the MCFD2-ERGIC-53 complex. They use NMR to identify an ER export motif that binds MCFD2 and also show that complex formation is enhanced by the presence of ERGIC-53. Many previous studies have reported the identification of various trafficking promoting motifs, but I think this is the most profound and thorough that I am aware of. However, as I said, the sole fact FVIII has an export motif that drives its ER export is neither novel nor surprising. Certainly, the most surprising finding of this paper is shown in Figures 4&5. EPO is a protein that is normally secreted and thus it is safe to assume it has its own ER export motif. I find it very surprising that the transplantation of the export motif (the passport sequence) enhances the secretion of EPO to a supra-physiologic level. The authors also nicely use the RUSH assay to further strengthen this notion. In my opinion, this is the main selling point of this work that would justify its publication in Nature Communications, but in its current form it requires some more additions and further work before this work can be accepted. Therefore, my recommendation is to accept this work pending the following revisions that I think are really essential to make this work a much nicer read for the scientific community:

1. Is glycosylation still needed for the “passport sequence” to exert its action. Does this sequence drive export of a glycosylation deficient mutant? This is a control experiment that would be nice to have, but is not as essential as the next experiment that I am asking for.

2. Does the “passport sequence” drive the export of a protein that normally does not leave the ER? One possibility would be to transplant it on a mutant protein that is unable to leave the ER such as the ATZ-mutant of alpha1-antitrypsin. Another possibility is to transplant it to an ER resident glycoprotein.

3. This image in Figure 5 indicates the EPO is a protein that traffics inefficiently. What about trafficking of other proteins? Is this export motif only working on proteins with inefficient trafficking?

Response to Revewers

Reviewer #1 (Remarks to the Author):

This manuscript reports the identification of a peptide sequence in coagulation factors V and VIII, which the authors propose serves as a "passport tag" to mark these proteins for efficient trafficking through the secretory pathway via interactions with the MCFD2/LMAN1 cargo receptor. They then go on to use this tag to boost the secretion of an unrelated protein, erythropoietin (EPO). Though the authors hypothesis is of potential interest, there are number of significant concerns as outlined below.

1) The authors focus on the N-terminus of the FVIII B-domain to identify their "passport" tag, citing studies from reference 20 that show that these sequences "influence the secretion level of FVIII." However, in reference 20 (Miao et al), though a significant increase in FVIII secretion is observed with addition of the first 54 aa of the B-domain, no further increase is observed with the addition of the next 63 aa (containing the current manuscript's "passport" sequence). How can the findings of the current manuscript be reconciled with these earlier studies, which seem to exclude a major effect of this putative "passport" sequence?

The first 54 amino-acid sequence of the B-domain in the construct contained the first seven residues (SDLLMLL) of our identified passport sequence. Its remaining three residues (RQS) reside in the following 63 amino-acid residues examined in reference 20. As pointed out by the reviewer, the first 54 amino-acid sequence, unlike the following 63 amino-acid sequence, significantly upregulated the secretion of FVIII. These results are consistent with the results of the present study showing that the functionally critical determinant of the passport sequence is the central leucine cluster (Fig. 4F and Supplementary Fig. 7 in the revised manuscript), which is entirely contained in the former sequence, whereas the following arginine residue, belonging to the latter, showed little or no contribution. We highlighted this point in the revised manuscript (p.7, lines 12–15).

2) Is this putative "passport" sequence uniquely conserved across species within the otherwise poorly conserved B-domain? Is the "passport" sequence also present in other putative MCFD2/LMAN1 cargoes, including alpha-1-antitrypsin and cathepsins C and Z? If not, how are these latter proteins targeted to LMAN1/MCFD2?

The MCFD2-binding motif corresponding to the passport sequence is highly conserved in FV and FVIII across species (Supplementary Fig. 1). MCFD2 also contributes to the efficient secretion of human galectin 3-binding protein (Fukamacji et al. Exp. Cell Res. 368:119–125, 2018). This glycoprotein shares a similar sequence, TDLLQLLLPR. We described this point in the revised manuscript (p.6, lines 16–19). In contrast, no such sequence is found in the lysosomal enzymes cathepsin Z and cathepsin C, which interact with ERGIC-53 in the early secretory pathway in an MCFD2-independent manner (Nyfeler et al. Traffic. 7:1473-81, 2006). Although an MCFD2-binding motif has not been unambiguously identified in alpha-1-antitrypsin, its plasma level slightly decreased in MCFD2 knockout mice in a sex-dependent manner (Zhu et al. Blood Adv. 2:1014-1021, 2018). This suggests that additional, unknown mechanisms underlie the transport of this cargo.

3) The authors rely on EPO as the sole experimental protein in which to test their hypothetical "passport" sequence. How generalizable is the effect of this passport sequence? Have the authors tried any other "typical" secreted proteins?

As per the review's comment, we examined the effect of the passport sequence on the secretion of soluble Fcγ receptor III (sFcγRIII), which is a secretory protein containing five N-glycans. We demonstrated that its secretion level was also significantly increased with the C-terminal tagged passport sequence. The data were added as Supplementary Fig. 4 in the revised manuscript. Accordingly, we revised the main text (p.6, line 20–p.7, line 1).

4) Are the dissociation constants observed for the factor VIII derived peptide with LMAN1/MCFD2 physiologically relevant?

It is assumed that ERGIC-53 captures an N-glycan displayed on a cargo glycoprotein, whereas MCFD2 interacts with a specific amino acid sequence (defined as passport sequence in this study). From the NMR titration data, the dissociation constant of the interaction between MCFD2 and the FVIII-derived peptide was estimated to be $1.1 \pm 0.1 \times 10^{-5}$ M. Further, we previously reported that the dissociation constant between the carbohydrate recognition domain of ERGIC-53 and its cognate high-mannose-type oligosaccharides is approximately 10^{-4} M (Kamiya et al. J. Biol. Chem. 283:1857–1861, 2008). Although the elementary interactions between ERGIC-53 and an FVIII glycan and between MCFD2 and its binding motif identified in this study are weak, their dual binding is likely to enhance the affinity of the cargo to be at physiologically relevant levels. We described these points in the revised manuscript (p.6, lines 13–16).

5) The authors show that deletion of the MCFD2–binding motif from factor VIIII decreases secretion from HCT116 cells (figure 4). Is the same effect seen with factor V? In figure 4E, the authors explore the effect of point mutations in the "passport" sequence on the efficiency of EPO secretion, localizing the most crucial function to the central leucine cluster. Is this conclusion supported by mutations of the same motifs within factor VIII and/or V?

As per the reviewer's comment, we created an FVIII mutant in which the central leucin cluster was replaced with five alanine residues. This mutant exhibited a significantly lower secretion level in comparison with the wild-type FVIII. The result has been described in the text (p.7, lines 12–15) and Supplementary Fig. 7 in the revised manuscript. We also attempted to produce a recombinant human FV in HCT116 cells. However, this was not successful despite extensive codon optimization. Because, to the best of our knowledge, there have been no reports describing recombinant production of FV, we are afraid that it is not hardly realized to establish an FV expression system within a reasonable timeframe.

6) the authors explain the smaller effect of ERGIC53 deletion on factor VIII secretion compared to MCFD2 by proposing overlap with the related protein ERGL. Does simultaneously deletion of both ERGL and ERGIC53 confirm this hypothesis?

As per the reviewer's comment, we generated the double-knockout cells of ERGL and ERGIC53 for the secretion assay of FVIII. As expected, FVIII secretion was further decreased in the double-knockout cells in comparison with either of the single-knockout cells. These data are shown as Fig. 4B (p.6, line 10).

7) In the experiments depicted in figure 4, were appropriate controls employed to normalize for variations in transfection efficiency?

According to the reviewer's suggestion, we presented the data including controls using transfection of GFP expression vector to normalize the transfection efficiency of individual cells (Fig. 4).

8) The authors might consider Endo-H treatment of cell lysates to examine the relative ER retention of the recombinant proteins under study.

According to the reviewer's comment, we conducted a western blotting analysis of the cell lysates with and without treatment with endoglycosidase H (Fig. R1 for review). The data indicated that wild-type EPO exhibited a higher level of accumulation in the cells in comparison with the

EPO-motif, consistent with their secretion levels. The intracellular EPO fraction contained significant quantities of its non-glycosylated form, which hampered the quantification of the pre-Golgi glycoforms.

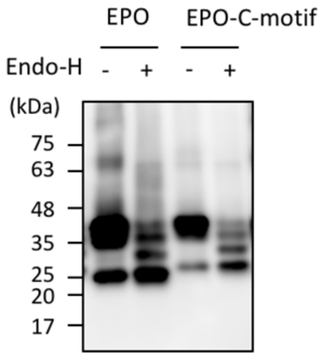

Fig. R1. Intracellular accumulation of EPO and the EPO-C-motif. Lysates of HCT116 cells transfected with EPO and the EPO-C-motif were purified using anti-flag conjugated resin and subjected to western blotting analysis using an anti-flag antibody.

Reviewer #3 (Remarks to the Author):

The manuscript by Yagi et al investigates the trafficking of FVIII and its regulation by the MCFD2-ERGIC-53 complex. They use NMR to identify an ER export motif that binds MCFD2 and also show that complex formation is enhanced by the presence of ERGIC-53. Many previous studies have reported the identification of various trafficking promoting motifs, but I think this is the most profound and thorough that I am aware of. However, as I said, the sole fact FVIII has an export motif that drives its ER export is neither novel nor surprising. Certainly, the most surprising finding of this paper is shown in Figures 4&5. EPO is a protein that is normally secreted and thus it is safe to assume it has its own ER export motif. I find it very surprising that the transplantation of the export motif (the passport sequence) enhances the secretion of EPO to a supra-physiologic level. In my opinion, this is the mainxselling point of this work that would justify its publication in Nature Communications, but in its current form it requires some more additions and further work before this work can be accepted. Therefore, my recommendation is to accept this work pending the following revisions that I think are really essential to make this work a much nicer read for the scientific community:

1. Is glycosylation still needed for the "passport sequence" to exert its action. Does this sequence drive export of a glycosylation deficient mutant? This is a control experiment that would be nice to have, but is not as essential as the next experiment that I am asking for.

In response to the reviewer's comment, we performed a secretion assay using mutationally deglycosylated EPO proteins. The wild-type EPO possesses three N-glycosylation sites, i.e., Asn24, Asn38, and Asn84. We prepared three kinds of N-glycan-deficient mutants (N24Q, N38Q, and N84Q). The data indicated that deglycosylation negatively affected the passport sequence-dependent secretion of EPO in a position-dependent manner. These data were added as Fig. 4E (p.7, lines 3–8).

2. Does the "passport sequence" drive the export of a protein that normally does not leave the ER? One possibility would be to transplant it on a mutant protein that is unable to leave the ER such as the ATZ-mutant of alpha1-antitrypsin. Another possibility is to transplant it to an ER resident glycoprotein.

As per the reviewer's comment, we conducted a secretion assay using the ATZ-mutant with or without the C-terminal passport sequence. We did not observe significant difference in the level of secretion (Fig. R2 for review). The data suggested that the passport sequence is unable to drive the export of this glycoprotein, at least with the conventional C-terminal tagging.

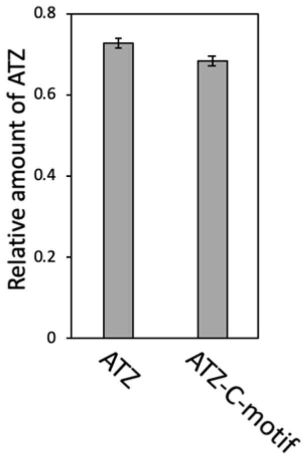

Fig. R2. Secretion level of the ATZ-mutant of alpha 1-antitrypsin with or without the C-terminal SDLLMLLRQS motif from HCT116 cells. The proteins were detected through an enzyme-linked immunosorbent assay using an anti-α1 antitrypsin antibody. Error bars represent the S.E.M. (n = 3 independent transfections).

3. This image in Figure 5 indicates the EPO is a protein that traffics inefficiently. What about trafficking of other proteins? Is this export motif only working on proteins with inefficient trafficking?

To address this issue, we examined the effect of the passport sequence on the secretion of soluble Fcγ receptor III (sFcγRIII), which is a secretory protein containing five N-glycans. As expected, its secretion level was also significantly increased with the C-terminal tagged passport sequence (Supplementary Fig. 3). The data were added as Supplementary Fig. 4 in the revised manuscript. Accordingly, we revised the paragraph in the text (p.6, line 20–p.7, line 1).

Reviewers' comments:

Reviewer #1 (Remarks to the Author):

The authors have provided a detailed response to the points raised in my and Reviewer 3's initial critiques, with the inclusion of additional data and corresponding revisions to the text. However, concerns remain as to the general applicability of their proposed simple 9 amino acid "passport sequence" to target secretory proteins to the LMAN1/MCFD2 cargo receptor.

The authors address our initial question #2 about evolutionary conservation of the putative passport sequence, and include supplemental figure 1 showing FV and FVIII alignments for a limited number of closely related mammalian species. Though not noted by the authors, the segment containing the factor VIII "passport sequence" is entirely absent/deleted from the more distant chicken and frog sequences. Though beyond the scope of the current manuscript, it would be interesting to know whether secretion of factor V and factor VIII in these latter species is no longer dependent of ERGIC-53/MCFD2.

A major issue raised in the initial review (point #3) was the question of how generalizable the effectiveness of this putative passport sequence might be when grafted onto other secreted proteins, with only EPO examined. In the revised manuscript, the authors added one additional control protein, sFcgammaRIII. Though these data do provide some support for the authors' hypothesis, the use of more than one such control, or a clear rationale for the choice of sFcgammaRIII would further strengthen the case. Did the authors test any additional protein cargoes that are not reported here? If so, such negative data should be included. In response to reviewer 3, the authors also examine alpha-1-antrypsin Z, as an example of a protein that is defective in exiting the ER, observing no effect for the passport sequence. The obvious control to include here would be wild-type alpha-1-antrypsin. Does the passport sequence effectively boost secretion of the latter?

In response to point #5 in the original review, questioning whether the same passport sequencer function can be demonstrated for factor V, the authors' noted technical difficulty producing recombinant factor V and state that to the best of their knowledge, "there have been no reports describing recombinant production of factor V…". However, a number of previous biochemical reports have focused on the analysis of recombinant FV for both wild type and mutant sequences. Indeed, a pubmed search identifies multiple such papers including several with "recombinant FV" in the title (eg. Biochemistry. 2004; 43:5803-10). A number of these groups successfully expressed functional FV by transient transfection in COS-1 cells. Nonetheless, this does seem to be a technical challenge for the authors, and I recognize that it might thus take significant additional work to address this point.

In response to point 6, the authors now analyze EPO–C secretion by cells deleted for both ERGL and ERGIC53, observing even greater reduction in factor VIII secretion than with either single knock-out, consistent with their proposed model of overlapping function between these proteins as cofactors for MCFD2. However, the double knockout appears to exhibit even lower factor VIII secretion than the MCFD2 knockout. Is this difference statistically significant? If so, how is this greater reduction in the double ERGL/ERGIC53 knockout explained?

Reviewer #3 (Remarks to the Author):

I have looked now at the revised version and at the response of the authors to my original comments. I still think that this is an interesting story. An important piece of new data are in the new supplementary Figure 3, where the authors show that the sFcγRIII secretion is enhanced by the passport sequence.

One thing is that I am not convinced that the "passport sequence" is a major regulator of secretion. Its effect is not transplantable to a non-secreted protein. It is also all too dependent on glycosylation. In this way, the glycosylation itself could also be called "passport sequence". However, these are pure semantics and does not affect the fact that the authors did identify a sequience element that potentiates secretion.

I have no further comments or requests.

Reviewer #1 (Remarks to the Author):

The authors have provided a detailed response to the points raised in my and Reviewer 3's initial critiques, with the inclusion of additional data and corresponding revisions to the text. However, concerns remain as to the general applicability of their proposed simple 9 amino acid "passport sequence" to target secretory proteins to the LMAN1/MCFD2 cargo receptor.

The authors address our initial question #2 about evolutionary conservation of the putative passport sequence, and include supplemental figure 1 showing FV and FVIII alignments for a limited number of closely related mammalian species. Though not noted by the authors, the segment containing the factor VIII "passport sequence" is entirely absent/deleted from the more distant chicken and frog sequences. Though beyond the scope of the current manuscript, it would be interesting to know whether secretion of factor V and factor VIII in these latter species is no longer dependent of ERGIC-53/MCFD2.

We appreciate the reviewer's insightful comment. We revised the multiple alignments of the passport sequences on FV and FVIII among animals including those of flog, chicken and zebrafish in Supplementary Fig.1 and modified the sentence (p.5, line 17). The passport sequences are not conserved in these non-mammalian species, indicating their FV/FVIII secretion systems do not depend on MCFD2.

A major issue raised in the initial review (point #3) was the question of how generalizable the effectiveness of this putative passport sequence might be when grafted onto other secreted proteins, with only EPO examined. In the revised manuscript, the authors added one additional control protein, sFcgammaRIII. Though these data do provide some support for the authors' hypothesis, the use of more than one such control, or a clear rationale for the choice of sFcgammaRIII would further strengthen the case. Did the authors test any additional protein cargoes that are not reported here? If so, such negative data should be included. In response to reviewer 3, the authors also examine alpha-1-antrypsin Z, as an example of a protein that is defective in exiting the ER, observing no effect for the passport sequence. The obvious control to include here would be wild-type alpha-1-antrypsin. Does the passport sequence effectively boost secretion of the latter?

As per the reviewer's comment, we examined possible effects of the passport tagging of α1-antrypsin (AAT) on its secretion from HCT116 cells. The secretion level of AAT with the C-terminal SDLLMLLRQS motif (AAT-C-motif) was not significantly elevated in comparison with

that of AAT. As suggested from the data shown in Figure 4F, validity of the passport sequence is restricted depending on its spatial arrangement with respect to the N-glycans displayed on cargos. While sFcγRIII possesses five N-glycosylation sites, ATT have only three, neither of which presumably occupied an effective position in terms of the carbohydrate/protein dual binding to the ERGIC-53/MCFD2 cargo receptor complex. We added the AAT secretion data (Supplementary Fig. 4B) in the revised manuscript and a remark on the aforementioned point (p.8, lines 8-11).

In response to point #5 in the original review, questioning whether the same passport sequencer function can be demonstrated for factor V, the authors' noted technical difficulty producing recombinant factor V and state that to the best of their knowledge, "there have been no reports describing recombinant production of factor V…". However, a number of previous biochemical reports have focused on the analysis of recombinant FV for both wild type and mutant sequences. Indeed, a pubmed search identifies multiple such papers including several with "recombinant FV" in the title (eg. Biochemistry. 2004; 43:5803-10). A number of these groups successfully expressed functional FV by transient transfection in COS-1 cells. Nonetheless, this does seem to be a technical challenge for the authors, and I recognize that it might thus take significant additional work to address this point.

We thank the reviewer for the extremely useful comments. Although we could not establish the recombinant FV expression system using HCT116 cells, we succeeded in producing it by use of COS-7 cells as suggested by the reviewer. Using this system, we examined effects of mutation of the passport sequence. Consequently, we revealed that replacement of the LLLL motif with AAAA in the FV passport sequence caused a significant reduction in its secretion from COS-7 cells. We presented these data as Supplementary Fig 7B with modifications of the text (p.5, lines 15-18).

In response to point 6, the authors now analyze EPO–C secretion by cells deleted for both ERGL and ERGIC53, observing even greater reduction in factor VIII secretion than with either single knock-out, consistent with their proposed model of overlapping function between these proteins as cofactors for MCFD2. However, the double knockout appears to exhibit even lower factor VIII secretion than the MCFD2 knockout. Is this difference statistically significant? If so, how is this greater reduction in the double ERGL/ERGIC53 knockout explained?

We appreciate the reviewer's constructive comments. Regarding the observed difference, we speculate that relative contributions of the N-glycans (recognized by ERCIC-53/ERGL) and the

passport sequence (recognized by MCFD2) to the interaction with the cargo receptor complex might be different for different cargos. Because FVIII possesses 25 N-glycans, it can multivalently interacts with an oligomeric form of ERGIC-53 (or ERGICL), thereby gaining a higher affinity for the cargo receptor. Although this may explain the greater contributions of the lectins in comparison with MCFD2 in the interaction with FVIII, we would refrain from describing such speculative discussion, which would be beyond the scope of this study focusing on the MCFD2-binding motif.

Reviewer #3 (Remarks to the Author):

I have looked now at the revised version and at the response of the authors to my original comments. I still think that this is an interesting story. An important piece of new data are in the new supplementary Figure 3, where the authors show that the sFcγRIII secretion is enhanced by the passport sequence.

One thing is that I am not convinced that the "passport sequence" is a major regulator of secretion. Its effect is not transplantable to a non-secreted protein. It is also all too dependent on glycosylation. In this way, the glycosylation itself could also be called "passport sequence". However, these are pure semantics and does not affect the fact that the authors did identify a sequience element that potentiates secretion.

I have no further comments or requests.

We thank the reviewer's insightful comment. As suggested from the data shown in Figure 4E, validity of the passport sequence is restricted depending on its spatial arrangement with respect to the N-glycans displayed on cargos. We described this point in the revised manuscript (p.8, lines 8-11).

REVIEWERS' COMMENTS:

Reviewer #1 (Remarks to the Author):

The authors have satisfactorily addressed all my comments. I just note one minor typo error in supp. fig 1, where "Flog" should be "Frog".

Reviewer #3 (Remarks to the Author):

I have looked at the manuscript again and maintain my initial reservation that I do not think that the so called passport sequence has any general relevance. The authors write in the response letter:

"validity of the passport sequence is restricted depending on its spatial arrangement with respect to the N-glycans displayed on cargos"

I think this is a weak argument for the validity of a general passport sequence.

The work is technically of really high quality. I also believe that the authors have discovered a sequence of importance for FVIII secretion. However, this sequence does not seem to be of any importance for general trafficking and not even for ERGIC-53 clients.

REVIEWERS' COMMENTS:

Reviewer #1 (Remarks to the Author):

The authors have satisfactorily addressed all my comments. I just note one minor typo error in supp. fig 1, where "Flog" should be "Frog".

Response: We thank the reviewer for pointing out this error. We have corrected it in the revised manuscript.

Reviewer #3 (Remarks to the Author):

I have looked at the manuscript again and maintain my initial reservation that I do not think that the so called passport sequence has any general relevance. The authors write in the response letter:

"validity of the passport sequence is restricted depending on its spatial arrangement with respect to the N-glycans displayed on cargos"

I think this is a weak argument for the validity of a general passport sequence.

The work is technically of really high quality. I also believe that the authors have discovered a sequence of importance for FVIII secretion. However, this sequence does not seem to be of any importance for general trafficking and not even for ERGIC-53 clients.

Response: According to the review's comments, we have modified our conclusions in the title, abstract (line 9) and text (p.8, line 7).
